# Homeobox transcription factor HbxA influences expression of over one thousand genes in the model fungus *Aspergillus nidulans*

**DOI:** 10.1371/journal.pone.0286271

**Published:** 2023-07-21

**Authors:** Sandesh S. Pandit, Jinfang Zheng, Yanbin Yin, Sophie Lorber, Olivier Puel, Sourabh Dhingra, Eduardo A. Espeso, Ana M. Calvo

**Affiliations:** 1 Department of Biological Sciences, Northern Illinois University, DeKalb, Illinois, United States of America; 2 Nebraska Food for Health Center, Department of Food Science and Technology, University of Nebraska, Lincoln, Nebraska, United States of America; 3 Toxalim (Research Centre in Food Toxicology), Université de Toulouse, INRAE, ENVT, INP-Purpan, UPS, Toulouse, France; 4 Department of Biological Sciences and Eukaryotic Pathogen Innovation Center, Clemson University, Clemson, South Carolina, United States of America; 5 Department of Cellular and Molecular Biology, Centro de Investigaciones Biológicas Margarita Salas (CSIC), Madrid, Spain; University of California Riverside, UNITED STATES

## Abstract

In fungi, conserved homeobox-domain proteins are transcriptional regulators governing development. In *Aspergillus* species, several homeobox-domain transcription factor genes have been identified, among them, *hbxA*/*hbx1*. For instance, in the opportunistic human pathogen *Aspergillus fumigatus*, *hbxA* is involved in conidial production and germination, as well as virulence and secondary metabolism, including production of fumigaclavines, fumiquinazolines, and chaetominine. In the agriculturally important fungus *Aspergillus flavus*, disruption of *hbx1* results in fluffy aconidial colonies unable to produce sclerotia. *hbx1* also regulates production of aflatoxins, cyclopiazonic acid and aflatrem. Furthermore, transcriptome studies revealed that *hbx1* has a broad effect on the *A*. *flavus* genome, including numerous genes involved in secondary metabolism. These studies underline the importance of the HbxA/Hbx1 regulator, not only in developmental processes but also in the biosynthesis of a broad number of fungal natural products, including potential medical drugs and mycotoxins. To gain further insight into the regulatory scope of HbxA in *Aspergilli*, we studied its role in the model fungus *Aspergillus nidulans*. Our present study of the *A*. *nidulans hbxA*-dependent transcriptome revealed that more than one thousand genes are differentially expressed when this regulator was not transcribed at wild-type levels, among them numerous transcription factors, including those involved in development as well as in secondary metabolism regulation. Furthermore, our metabolomics analyses revealed that production of several secondary metabolites, some of them associated with *A*. *nidulans hbxA*-dependent gene clusters, was also altered in deletion and overexpression *hbxA* strains compared to the wild type, including synthesis of nidulanins A, B and D, versicolorin A, sterigmatocystin, austinol, dehydroaustinol, and three unknown novel compounds.

## Introduction

Developmental studies of the model filamentous fungus *Aspergillus nidulans* have provided broad valuable insight into the genetic regulatory mechanisms of morphogenesis in fungi [1–3]. *A*. *nidulans* efficiently disseminates by asexual reproduction, forming specialized structures called conidiophores, which bares large numbers of air-borne conidia. Activation of conidiogenesis is mediated by several transcription factor genes, including *flb* genes, such as *flbB*, *flbC*, *flbD*, and *flbE*, which activate the central regulatory pathway comprised of *brlA*, *abaA* and *wetA* [4]. This model organism is also able to reproduce sexually by producing cleistothecia, fruiting bodies containing meiospores called ascospores. Cleistothecia form by aggregation of vegetative mycelia, surrounded by nursing Hülle cells. This results in the formation of cleistothecial primordia, which later mature into melanized cleistothecia [5, 6]. Several genes are involved in the regulation of these processes, including *nsdD*, *medA*, *phoA*, *stuA*, *lsdA* and *tubB* [7–12].

Other developmental regulators include Homeobox-domain transcription factors (HD-TFs). These are global regulators governing developmental processes in many eukaryotic organisms [13–15]. The HD contains approximately 66 conserved amino acid that bind to the promoter of genes governing development and other cellular processes in fungi, plants, and animals. In general, fungi possess 6–12 HD-TF genes in their genome [16, 17]. The first reported HD-FT gene is *pah1* in *Podospora anserina*, where it was shown to control microconidiation as well as mycelial branching [18]. Loss-of-function of seven HD-TF genes in this fungus revealed their role in sexual development [19]. Another study showed that several HD-TFs in the rice pathogen *Magnaporthe oryzae* are necessary for proper hyphal growth, asexual development, and appressorium formation [20, 21]. In three species of *Fusarium*, loss-of-function of the *htf1* homeobox gene leads to alteration of phialides during conidiophore formation, accompanied by a drastic reduction in conidial production [22]. In the fungus *Botrytis cinerea*, the BcHOX8 gene has been shown to regulate growth, conidiation, and virulence in different host plants [16]. Also, lack of the *GRF10* HD-TF gene in the human pathogen *Candida albicans* resulted in a decrease in growth, defects in chlamydospore morphology, alterations in biofilm production, and a reduction of virulence [23].

HD-TFs are also key regulators in species of the genus *Aspergillus*. In the agriculturally relevant fungus *Aspergillus flavus*, deletion of eight HD-FT genes revealed that *hbx1* in particular, was required for normal vegetative growth and production of conidia and sclerotia [17]. The regulation of morphological development as well as regulation of secondary metabolism (SM), are often genetically linked [24–26]. Interestingly, in this case also, the production of secondary metabolites, including mycotoxins (aflatoxins, cyclopiazonic acid and aflatrem), was under the regulation of *hbx1* [17]. Furthermore, study of the *hbx1-*dependent transcriptome indicated its importance in morphological development and in regulation of secondary metabolite production [27]. Remarkably, the gene category corresponding to SM was the most affected by *hbx1*. Additionally, in our previous study of the *hbx1* homolog in the opportunistic human pathogen *Aspergillus fumigatus*, *hbxA*, showed that this gene is necessary for proper spore formation, regulating expression of *brlA*, *flbB*, *flbD* and *fluG* [28]. The *hbxA* gene also influenced germination rate and virulence in a neutropenic mouse model. Interestingly, as in the case of *A*. *flavus*, *A*. *fumigatus hbxA* affected production of various secondary metabolites, including fumigaclavines, fumiquinazolines, compounds that accumulate in asexual structures, whose production is linked to *brlA* expression [29–31], and chaetominine, an alkaloid compound that is being tested to combat leukemia cells [32]. Both *A*. *flavus* and *A*. *fumigatus* studies indicate that HbxA/Hbx1 is a global regulator of SM in these fungi, in addition to its role in morphogenesis. We hypothesize that this would also be the case in other *Aspergillus* species. HbxA also affects *A*. *nidulans* conidiation [33, 34] in a similar manner as that in *A*. *flavus* and *A*. *fumigatus* [17, 27, 28]. To gain further insight into the regulatory scope of *hbxA* in the genus *Aspergillus*, in the present study, we characterized its role in the model fungus *A*. *nidulans* by transcriptome and metabolomics approaches. Our findings indicate that more than one thousand genes were differentially expressed in the absence of this regulator or when it was over-expressed, as compared to the wild type. These include several transcription factor genes, including those involved in development and SM production. Our study revealed that numerous secondary metabolites gene clusters are *hbxA*-dependent in *A*. *nidulans*. Furthermore, our analyses also indicated that *A*. *nidulans* metabolome is affected by *hbxA*, including production of some unknown novel compounds.

## Materials and methods

### Phylogenetic analysis

Deduced amino acid sequences of HbxA homologs were obtained from FUNGIDB (https://fungidb.org/fungidb/) website. BLASTp was performed against the protein sequence database (pdb). Percentage (%) similarity was found using Pairwise sequence alignment using EMBOSS Needle (ebi.ac.uk/Tools/psa/emboss_needle/). The phylogenetic tree was constructed using MEGA v6.0 and the Maximum Likelihood model with bootstrap value of 1000.

### Strains used and culture conditions

The *A*. *nidulans* strains used in this study are listed in Table 1. Strains were grown on glucose minimal medium (GMM) [35] with appropriate supplements for their respective auxotrophic markers [35]. For solid medium, agar (15 g/L) was added. Strains were stored as 30% glycerol stocks at -80°C.

**Table 1:** Strains used in this study.

Strain Name	Pertinent Genotype	Source
RMJP1.49	*pyrG89; argB2; Δnku*::*argB; pyroA4*	[36]
TSSP38.1	*pyrG89; argB2; Δnku*::*argB; pyroA4;ΔhbxA*::*pyrG;pyroA*	This Study
TSSP40.1	*pyrG89; argB2; ΔnkuA*::*argB; pyroA4; ΔhbxA*::*pyrG*, *hbxA*::*pyroA*	This Study
TSSP34.1	*pyrG89; argB2; ΔnkuA*::*argB; pyroA4; gpdA*(p)::*ΔhbxA*::*trpC*(t)::*pyrG; pyroA*	This Study
TRV50.2	Wild type	[37]

### Generation of the *hbxA* deletion strain (Δ*hbxA*)

The DNA cassette employed to obtain the deletion *hbxA* strain (TSSP38.1) was generated by fusion polymerase chain reaction (PCR) through a previously described method [38]. All primers used in this study are listed in Table 2. The 1.5 kb 5`UTR region of the *hbxA* locus was PCR amplified using P#2154/SD3 and P#2155 primers from genomic DNA of the *A*. *nidulans* FGSC4 wild-type strain. Similarly, the 1.1 kb 3`UTR of *hbxA* was amplified using P#2156 and P#2157 primers also from genomic DNA. The 1.9 kb *A*. *fumigatus pyrG* selectable marker was amplified from plasmid p1439 [39] using P#2158 and P#2159 primers. The 5`and 3`UTR fragments were fused to the selectable *pyrG* marker using P#2160/SD9 and P#2161 primers. The resultant fusion product was transformed into RMJP1.49 strain using a polyethylene glycol mediated protocol as described previously [38]. Transformants were confirmed by diagnostic PCR using P#2154 and P#963 primers. The selected deletion *hbxA* strain was then transformed with a DNA fragment containing the *A*. *nidulans pyroA* gene, PCR amplified with primers P#1042 and P#1045 from genomic DNA, resulting in strain TSSP38.1.

**Table 2:** Primers used in this study.

Name	Sequence (5’ → 3’)
P#2154/SD3	CCCGCTGATGTATGGTGAGGC
P#2155	TGGTGTAGGATGCGATGCGG
P#2156	CATCTCCTCCTTCAACACCAGGG
P#2157	GGTCTGAGGTCTTGCCGTTTCC
P#2158	CCGCATCGCATCCTACACCAACCGGTCGCCTCAAACAATGCTCT
P#2159	CCCTGGTGTTGAAGGAGGAGATGGTCTGAGAGGAGGCACTGATGCG
P#2160/SD9	CGCTCCCTTGAAACTCCGAGAG
P#2161	CACAGTAGGCACGAATGGCGTT
SD1	ACCGGTCGCCTCAAACAATGCTCT
SD2	GTCTGAGAGGAGGCACTGATGCG
SD4	GCGTTTTATTCTTGTTGACATGGGGTCCCTTAGCCGAAATTGGTGGG
SD5	CCCATGTCAACAAGAATAAAACGC
SD6	CCGAGTGGAGATGTGGAGT
SD7	ACTCCACATCTCCACTCGGGCCCATATCTTCCGTAGCAGTC
SD8	AGAGCATTGTTTGAGGCGACCGGTGACGGAGAGCTGAGAGTCCTAG
SD10	CAGAGCACCGCCGTGGTATTG
P#2962	GTCTCGTAGGTCTCTTGACGACCG
P#2238	AAAAAAGGCGCGCCATGAATTATATCCATCATCCATACCCTTTCGCTG
P#2239	AAAAAAAAAAGCGGCCGCTTAGCCGAAATTGGTGGGGGTC
P#1042	GCCGAAAAGGACCACGAATACCCGC
P#1045	CACCGCCAACGGAGACAATCAAGCC
P#963	GAGCAGCGTAGATGCCTCGAC
P#2093	GACCTAATACAGCCCCTACAACGACC
P#2218	GCGGCCGCTTAGCCGAAATTGGTGGGGGTC

### Generation of the *hbxA* complementation strain (*hbxA*-com)

The complementation strain (TSSP40.1) was generated by re-introducing the wild-type *hbxA* allele into the *ΔhbxA* strain at the same locus. The complementation cassette was generated as follows: first, a DNA fragment containing the *hbxA* coding region and a 3.7 kb 5’UTR was PCR amplified using P#2154/SD3 and SD4, and the *trpC* terminator fragment was amplified with primers SD5 and SD6 using *A*. *nidulans* genomic DNA as a template. The *A*. *fumigatus pyroA* gene (Afub_055620) was amplified from genomic DNA using primers SD7 and SD8. *A*. *fumigatus pyrG* was amplified from plasmid p1439 [38] using primers SD1 and SD2. All four PCR fragments were fused together using primers P#2160/SD9 and SD10 in a single reaction using Prime Star DNA polymerase (Clonetech, USA). The resulting fusion product was then transformed into the *hbxA* deletion strain (TSSP38.1) using methods previously described [38]. Fungal transformants were confirmed using diagnostic PCR with primers P#2154 and P#2962.

### Generation of the *hbxA* overexpression strain (OE*hbxA*)

To generate the over-expression *hbx1* strain (TSSP34.1), the coding region of *hbxA* was first amplified from *A*. *nidulans* genomic DNA using P#2238 and P#2239 primers. The resulting PCR product was digested with *Asc*I and *Not*I and ligated to pTRS2 plasmid, previously digested with the same enzymes. pTRS2 contains the *gpdA* promoter, *gpdA*_(p)_, and *trp*C terminator, *trp*C_(t)_. The resulting plasmid, pSSP34.1, was transformed into the *A*. *nidulans* RJMP1.49 strain, and transformants were screened by PCR using P#2093 and P#2218 primers. The selected overexpression *hbxA* strain was then transformed with a DNA fragment containing the *A*. *nidulans pyroA* gene, PCR amplified with primers P#1042 and P#1045 from genomic DNA, resulting in strain TSSP34.1.

### Transcriptome analysis

#### RNA purification and sequencing

Plates containing 25 mL of solid GMM with the appropriate supplements were top-agar inoculated with 5 mL of medium containing ~5 x10^6^ spores/mL of wild-type (WT) control, Δ*hbxA*, *hbxA-*com or OE*hbxA* (Table 1). Cultures were incubated in the dark at 37°C. After 72 h of incubation, mycelia were collected, frozen in liquid nitrogen, and lyophilized. Total RNA was extracted from mycelia using an RNeasy Plant Mini Kit (Qiagen, Germantown, Maryland, USA) following the manufacturer’s protocol. RNA was further purified using Dynabeads mRNA Purification Kit (Thermo Fisher Scientific Inc., Massachusetts, USA). RNA quality was assessed using an Agilent Bioanalyzer. Sequencing was performed as a HiSeq 2000 single read 1x100bp lane. The experiment was carried out with 3 biological replicates.

#### Read mapping, decontamination and read count

The RNA reads were trimmed by trim_galore [40] with the default parameter. Kraken2 [41] was run on trimmed reads to check the contamination. Then, reads were mapped to reference genome downloaded from FungiDB (*Aspergillus nidulans* FGSC4) [42]. Unmapped reads were removed to get clean reads. The clean reads were then repaired to pair-end reads with BBTools [43]. These final clean pair-end reads were remapped to reference genome again using hisat2 [44].

Mapped reads in SAM format were sorted by coordinates with samtools [45] to obtain the BAM format mapped reads. Then read count and TPM (Transcripts Per Kilobase Million) were calculated by running StringTie [46] and python script. The parameters were set not to infer new transcripts with the reference gene annotation file (also downloaded from FungiDB).

#### Differentially expressed coding genes (DEGs)

The read counts table was used as input for DEseq2 [47]. This package was used to determine DEGs by comparing read counts between two strains. Significant up regulated genes were determined with -log10 q-value < = 2 and log2 fold change > = 2, while significant down regulated genes were defined with -log10 q-value < = 2 and log2 fold change < = -2. Control vs. OE*hbxA* and Control vs. Δ*hbxA*. python script was developed to convert gene id between FungiDB and FungiFun2 so that the webserver of FungiFun2 can be used to perform FunCat term annotation and enrichment of DEGs for Control vs. OE*hbxA* and Control vs. Δ*hbxA* [48]. Heat maps of TPM (transcript per million) values of DEGs of secondary metabolism clusters were calculated by averaging all TPM values of all replicates.

Evaluation of differentially expressed ortholog genes in *A*. *nidulans* and *A*. *flavus* was carried out by using the MCL algorithm in combination with all-versus-all protein BLAST search, similar to a method previously described [49]. Proteins with BLAST hits were filtered with the following parameters: 1, query and subject coverage is greater than 60%. 2, e-value is less than 1^−5^. 3, the percent of identity is greater than 60%. And then, the filtered hits were fed into OrthoMCL with an inflation parameter of 2 to generate orthogroups between these two species.

To analyze changes in the expression of genes in secondary metabolite biosynthetic gene clusters (SMGs), 67 SMGs were extracted [50]. SMGs expression related figures were plotted with python seaborn package. In addition, expression of 521 transcript factors (TFs) was also analyzed. The list of TFs and their function annotations were derived from a previous report [51].

### Metabolomics

#### Thin- layer chromatography

Wild-type control, Δ*hbxA*, *hbxA-*com, and OE*hbxA* were top-agar inoculated with 5 mL of medium containing ~5 x10^6^ spores/mL on solid GMM and grown at 37°C for 3 days. Three 16-mm diameter cores per plate were collected and extracted with chloroform. Overnight dried extracts were resuspended in 200 μL chloroform. Sample were separated using thin-layer chromatography (TLC) as previously described [52] on silica gel plates using benzene and glacial acetic acid [95:5(*v*/*v*)] as solvent system. Aluminum chloride (15% in ethanol) was then sprayed, and plates were baked for 10 min at 80°C. Bands were visualized under UV light (375 nm). Sterigmatocystin (ST) standard was purchased from Sigma-Aldrich (St. Louis, MO, USA).

#### Analysis of secondary metabolites by liquid chromatography combined with mass spectrometry (LC-MS)

Chloroform extracted samples were also analyzed by LC-MS. Samples were resuspended in 500 μL of acetonitrile/water (50:50, v/v), shaken vigorously for 30 s and then treated with a sonicator (Bransonic 221 Ultrasonic bath, Roucaire, Les Ulis, France) for 2 h. A volume of 250 μL of pure ACN was added to each sample, followed by vigorous shaking (30s) and centrifugation (pulse). Secondary metabolites analysis was performed using Acquity ArcSystem HPLC (Waters, Saint-Quentin-en-Yvelines, France) combined with an LTQ Orbitrap XL high-resolution mass spectrometer (Thermo Fisher Scientific, Les Ulis, France). A volume of 10 μL of the suspension was injected into a reversed-phase 150 mm × 2.0 mm, Luna® 5 μm C18 column (Phenomenex, Torrance, CA, U.S.A.). Water acidified with 0.1% formic acid was used as phase A and 100% acetonitrile was used as phase B with the following elution gradient: 0 min 20% B, 30 min 50% B, from 35 to 45 min 90% B, from 50 to 60 min 20% B at 30°C at a flow rate of 0.2 mL min^-1^. HRMS acquisitions were achieved with electrospray ionization (ESI) in positive and negative modes, as previously reported [28]. MS/MS spectra were obtained with CID mode at low resolution and collision energy of 35%.

### Statistical analysis

Statistical analysis was applied to analyze all quantitative data in this study utilizing analysis of variance (ANOVA) in conjunction with a Tukey multiple-comparison test using a *p* value of <0.05 for samples that are determined to be significantly different.

## Results

### HbxA is conserved in numerous fungal species

**Fig 1:**
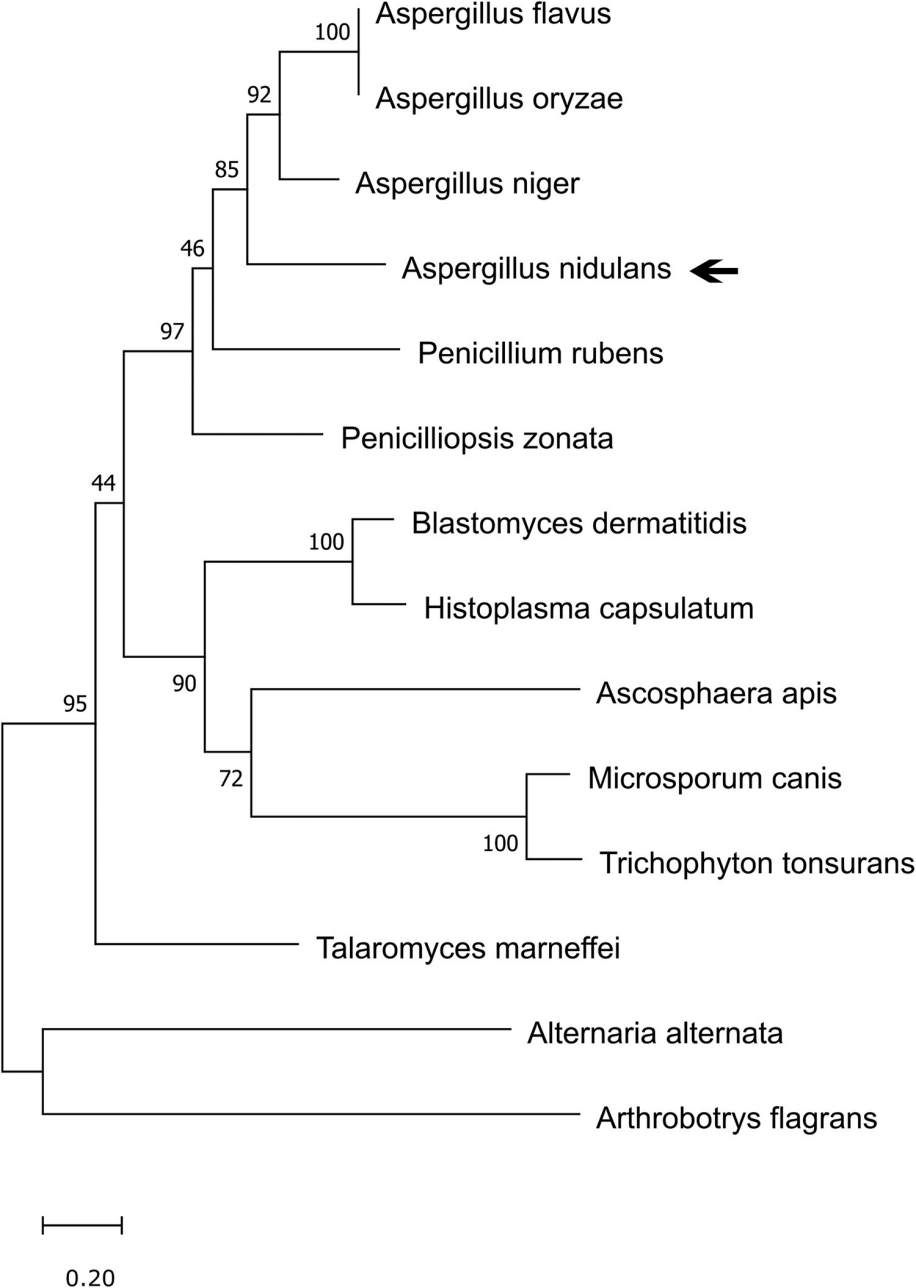
Phylogenetic analysis of *Aspergillus nidulans* HbxA. The phylogenetic tree was constructed using MEGA v6.0 and the Maximum Likelihood model with bootstrap value of 1000 (http://megasoftware.net/).

Our phylogenetic analysis confirmed that the *hbxA* deduced amino acid sequence corresponds to a transcription factor containing a homeodomain (S1 Fig). HbxA homologs are present in other *Aspergillus* species, including *A*. *flavus* [17, 27], *A*. *fumigatus* [28], *Aspergillus niger* and *Aspergillus terreus* (Fig 1, S1 Fig and Table 3), as well as in species of other fungal genera, such as *Alternaria alternata*, *Arthrobotrys flagrans*, *Ascosphaera apis*, *Blastomyces dermatitidis*, *Histoplasma capsulatum*, *Microsporum canis*, *Penicilliopsis zonata*, *Penicillium rubens*, *Talaromyces marneffei* and *Trichophyton tonsurans* (Fig 1, S1 Fig and Table 3). Of the sequences analyzed, *A*. *niger* HbxA was the closest homolog to *A*. *nidulans* HbxA, with 56.40% identity and 68.4% sequence similarity.

**Table 3:** Phylogenetic analysis of *A*. *nidulans* HbxA and homologs in other fungal species. HbxA homologos were retrieved from FUNGIDB website and BLASTp was performed against protein sequence database. % similarity was found utilizing Pairwise sequence alignment using *A*. *nidulans* HbxA as search query against each protein of interest using EMBOSS Needle.

Species (sorted)	Identity% (Needle)	Similarity% global Pairwise alignment
*Aspergillus niger*	56.4	68.4
*Aspergillus flavus*	52.9	65.2
*Penicilliopsis zonata*	44.5	54.3
*Penicillium rubens*	43.7	56.9
*Talaromyces marneffei*	37.1	49.3
*Blastomyces dermatitidis*	36.9	49.9
*Histoplasma capsulatum*	36.9	49.5
*Aspergillus terreus*	35.5	46.4
*Microsporum canis*	34.0	43.7
*Trichophyton tonsurans*	32.3	44.9
*Ascosphaera apis*	30.3	41.8
*Arthrobotrys flagrans*	21.5	34.3
*Alternaria alternata*	20	31.5

### *hbxA* is required for normal development in *A*. *nidulans*

**Fig 2:**
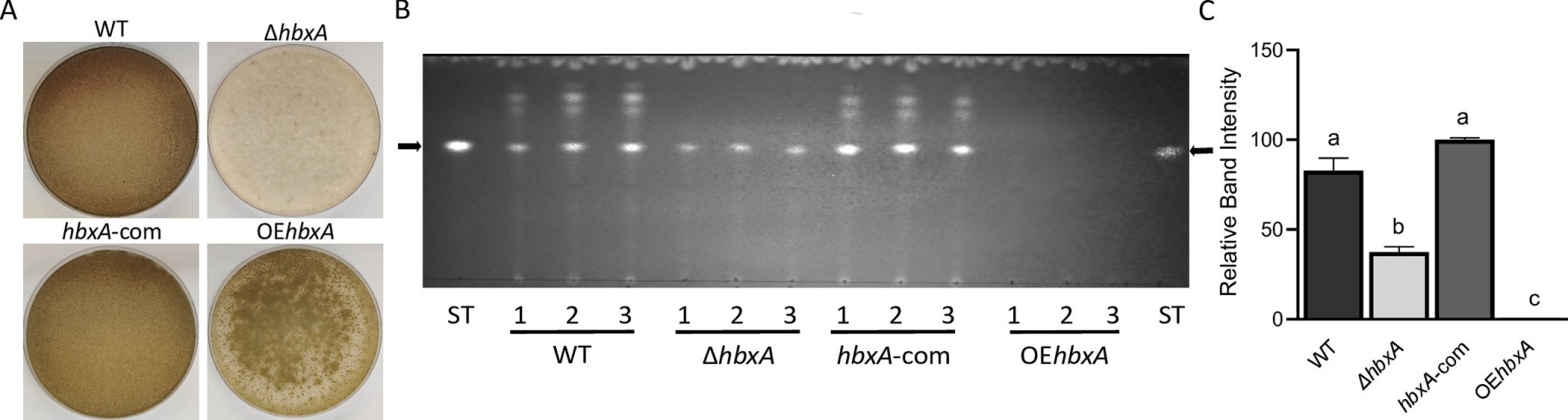
*hbxA* is required for normal conidiation and for production of ST and other secondary metabolites in *A*. *nidulans*. **(A)** Cultures of wild type, deletion, complementation and overexpression *hbxA* strains, top-agar inoculated on glucose minimum medium (GMM) and incubated for 7 days in the dark at 37°C. **(B)** TLC analysis of extracts from top-agar inoculated cultures incubated for 3 days in the dark. Black arrows indicate ST standard. The experiment was carried out with three replicates. **(C)** Densitometry of TLC analysis of ST levels in (B). The densitometry was performed using the http://biochemlabsolutions.com/GelQuantNET.html website. Error bars represent the standard error. Columns of different letters represent values that are statistically different *p* value of <0.05.

To determine the regulatory scope of *hbxA* in *A*. *nidulans*, three strains were generated, a deletion strain, Δ*hbxA*, a complementation strain, *hbxA-*com, and an over-expression strain, OE*hbxA* (S2 Fig). Deletion, complementation and overexpression strains were confirmed by diagnostic PCR, yielding the expected 3.01 kb PCR product for Δ*hbxA*, a 3.96 kb DNA fragment for *hbxA-*com and a 3.16 kb DNA fragment for OE*hbxA*. Our results confirmed that absence of *hbxA* results in a drastic reduction of conidiation (Fig 2A), as previously shown [33, 34].

### *hbxA* regulates secondary metabolism

Our TLC analysis indicated that deletion of *hbxA* reduces sterigmatocystin (ST) production in *A*. *nidulans* by approximately 50% when compared with levels in the wild-type strain (Fig 2B and 2C). Importantly, overexpression of *hbxA* completely blocked ST production. Additionally, synthesis of other metabolites was also affected by deletion or forced overexpression of *hbxA* compared to the control strain. The absence of metabolites was particularly notable in the OE*hbxA* strain extracts. These results suggested that the regulatory role of *hbxA* is broader than originally expected, controlling not only developmental processes but also acting as a global regulator of secondary metabolism.

### *hbxA*-dependent transcriptome in *A*. *nidulans*

#### More than one thousand genes are regulated by *hbxA* in *A*. *nidulans*

**Fig 3:**
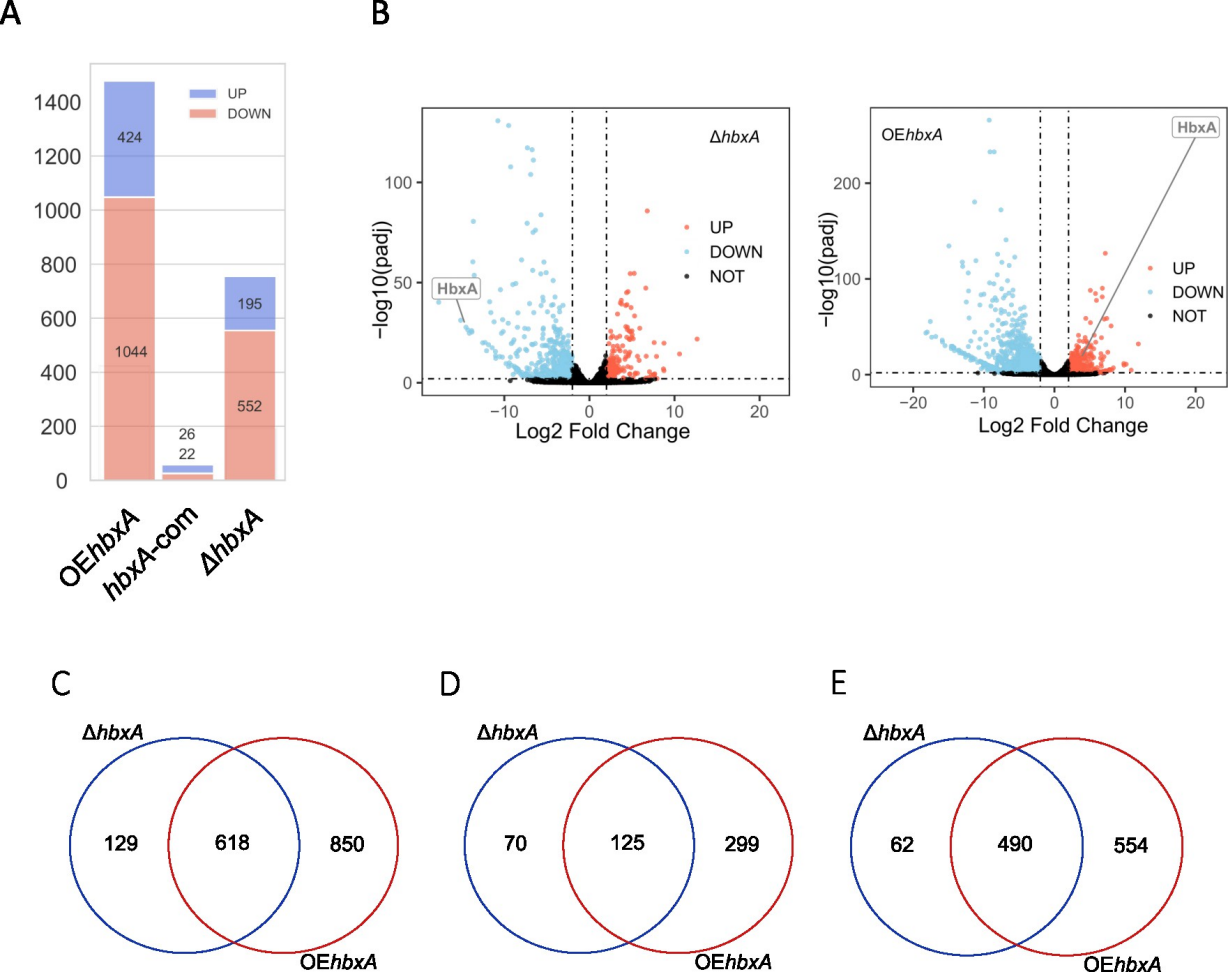
Number of DEGs in *A*. *nidulans* when expression of *hbxA* is altered by *hbxA* deletion or overexpression. **(A)** Number of significantly upregulated (purple) and significantly downregulated (orange) DEGs estimated by DeSeq2. **(B)** Volcano plot of log2 fold change vs. -log10 q-value of all the genes in Δ*hbxA*, and OE*hbxA* vs. control. Significantly upregulated genes are shown as red dots, significant down regulated genes are shown as blue dot and other genes are shown as black. The x-axis represents the log2 of the fold change determined by DeSeq2. The y-axis is the log10 of the adjusted p-value from DeSeq2. The cut offlog10 fold change value to determine the upregulated expression is greater than 2 while -2 is for down regulated expression. The -log10 q-value cutoff was set to 2 to determine the significant expression or not. **(C-D)** Venn Diagrams showing the overlap of DEGs in Δ*hbxA* and OE*hbxA* **(C)**, and the overlap of upregulated **(D)** and downregulated DEGs **(E)** in Δ*hbxA* and OE*hbxA*. Venn Diagrams were constructed using https://bioinformatics.psb.ugent.be/cgi-bin/liste/Venn/calculate_venn.htpl website.

RNA-sequencing analysis revealed that of the predicted 11286 genes present in *A*. *nidulans* genome [53], 552 were downregulated, and 195 were upregulated in the Δ*hbxA* strain compared with the wild-type control strain (S1 Table, Fig 3). Over-expression of *hbxA* resulted in an even more pronounced effect on the *A*. *nidulans* transcriptome, where 1044 genes were downregulated, and 424 genes were upregulated in the OE*hbxA* strain in comparison to the wild type. In strong contrast, the comparison of the complementation strain and wild type showed that the two strains present very similar expression patterns. Expression of 618 genes in the *A*. *nidulans* genome was altered by either deletion or overexpression of *hbxA*, many of them presenting the same expression pattern of upregulation or downregulation when *hbxA* was either deleted or overexpressed (Fig 3).

### Comparison of *hbxA/hbx1* DEGs in *A*. *nidulans* and *A*. *flavus*

**Fig 4:**
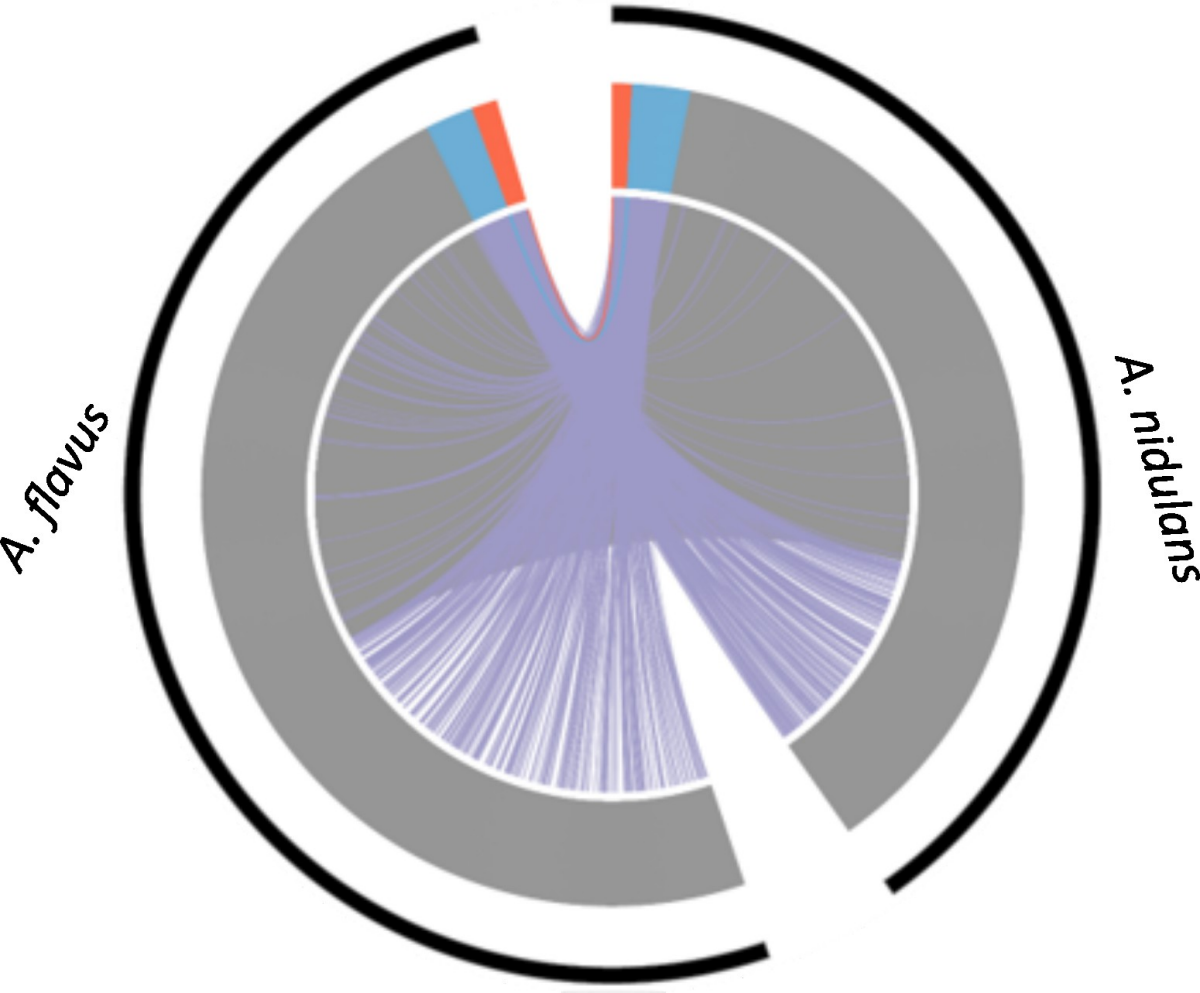
Comparison of orthologous genes affected by deletion of *hbxA* in *A*. *nidulans* and *A*. *flavus*. Both upregulated orthologous genes were colored in red. Both downregulated orthologous genes were colored in blue. No expression changed orthologous genes are colored in grey. Two orthologous genes having different regulation status are colored in purple. The significantly regulated genes were defined as |log2 fold change| < = 2 and q-value < = 0.05.

The comparison of the current *A*. *nidulans hbxA*-dependent transcriptome study with the previous *A*. *flavus hbx1* results [27] is shown in Fig 4. Only a small percentage of homologs were differentially expressed in the absence of *hbxA* and *hbx1* in *A*. *nidulans* and *A*. *flavus*, respectively, with respect to the corresponding wild types. Most of the DEGs in *A*. *nidulans* are not DEGs in the *A*. *flavus* study.

### Expression of numerous TF genes is *hbxA-*dependent in *A*. *nidulans*

**Fig 5:**
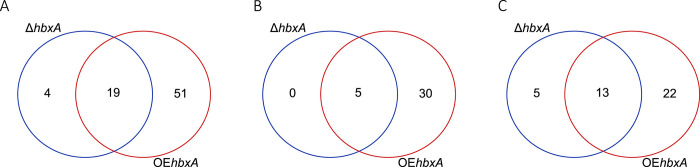
Number of transcription factor (TF) genes controlled by *hbxA in A*. *nidulans*. **(A)** Venn Diagram showing the overlap of differentially expressed TF genes in Δ*hbxA* and OE*hbxA*. **(B-C)** Venn Diagrams showing the overlap of upregulated **(B)** or downregulated **(C)** TF genes in Δ*hbxA* and OE*hbxA*. Venn Diagrams were constructed using https://bioinformatics.psb.ugent.be/cgi-bin/liste/Venn/calculate_venn.htpl website.

Based on our analysis, 74 out of 521 TFs genes in *A*. *nidulans* [51] were regulated by *hbxA* under the culture conditions assayed (S2 Table). Some of these differentially expressed TF genes also presented the same expression pattern of upregulation or downregulation when *hbxA* was either deleted or overexpressed (Fig 5).

Our results indicated that overexpression of *hbxA* caused upregulation of developmental regulators, including genes of the central developmental pathway, *brlA*, *abaA* [54–57], *fluffy* genes *flbC* and *flbD* [58], and another HD-TF gene, *hbxB*, that regulates asexual and sexual development in *A*. *nidulans* [34]. In addition, the developmental regulatory gene *zcfA* [59] was also upregulated by *hbxA* overexpression. Some of the upregulated TFs genes in OE*hbxA* are involved in both governing development as well as SM, such as the master transcription factor *mtfA* [37], *urdA*, [60], *sclB* [61], *osaA* [62], and *velB* [63]. Other upregulated *hbxA*-dependent TF-DEGs annotated to be putatively involved in SM regulation include AN8391 and AN6788. Other upregulated TF genes have an important role in primary metabolism, such as *glcD*, which has a putative role in protein dimerization and activation of *areB*, [64], *galR*, which is known to regulate the D-galactose catabolic pathway [65] and *creA* repressor of carbon catabolite [66]. Other upregulated TF genes were *rfeC*, whose ortholog in *Saccharomyces cerevisiae* promotes *FLO11* expression [67], the *mcnB* fork-head like transcription factor [68], as well as expression of some other uncharacterized putative transcription factors genes (S2 Table).

Overexpression of *hbxA* in *A*. *nidulans* caused downregulation of other developmental genes such as *fhpA*, with a role in sexual development [69], *mat1*, involved in activation of the alpha-domain mating-type protein [70]. Overexpression of *hbxA* also caused downregulation of *metZ*, a transcription factor involved in the regulation of sulfur metabolism [71]. TF genes AN8377, AN8645, AN3385 and AN8918 predicted to be involved in SM, are also downregulated in this strain (S2 Table).

Interestingly, deletion of *hbxA* also resulted in an increase in the expression of *brlA*, *abaA*, and *urdA*, as in the case of OE*hbxA*. It also increased the expression of *tah-3*, which is involved in conidiophore development and tolerance for harsh environment [72] (S2 Table). Deletion of *hbxA* also upregulated *veA* (S1 Table). The *veA* gene product, VeA, which contains a NF-κ-B like DNA-binding domain [73], is well known as a global regular that interacts with at least nine other proteins, LlmF, VapA, VipA, VipC, VelB, MpkB, FphA, LreB and LaeA [74], governing several signaling pathways and consequently multiple cellular processes, including development and SM [25].

Absence of *hbxA* in *A*. *nidulans* downregulated the expression of various transcription factors, including the gene encoding the alpha-domain mating-type protein, *mat1* [70], as in overexpression of *hbxA*. Deletion of *hbxA* also showed downregulation of *metZ*, involved in methionine biosynthesis [75] the nitrogen-dependent *mdpE*, which regulates production of a secondary metabolite called monodictyphenone [76]. The putative SM TF gene AN4933 is downregulated, and AN3385, AN8645 and AN8918 are also downregulated in deletion *hbxA*, as in OE*hbxA*.

### *hbxA* affects the expression of genes in SM gene clusters and biosynthesis of natural products in *A*. *nidulans*

**Fig 6:**
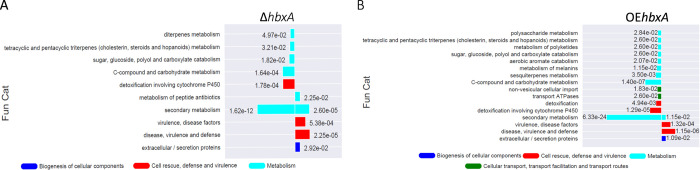
FunCat enrichment of significant DEGs found in (A) Δ*hbxA* and (B) OE*hbxA* vs. control. The -log10 of the q-value of DEGs in each term is proportional to the length of the bars. FunCat annotations and q-value is determined by FungiFun2 webserver. Downregulated genes are to the left of the origin and up regulated genes to the right.

Our TLC analysis revealed that both deletion and overexpression of *hbxA* negatively affect ST production (Fig 2B and 2C) as well as the production of other secondary metabolites. Furthermore, FunCat enrichment analysis revealed that differentially regulated genes in the Δ*hbxA* versus wild type and OE*hbxA* versus wild type comparisons have significant functional overlap (Fig 6). DEGs genes are dramatically enriched for secondary metabolism-related processes for both; most of those genes are downregulated when *hbxA* is either deleted or overexpressed, particularly in the latter. Other categories showing enrichment include disease, virulence, and defense; virulence disease factors; C-compound and carbohydrate metabolism; and detoxification.

**Fig 7:**
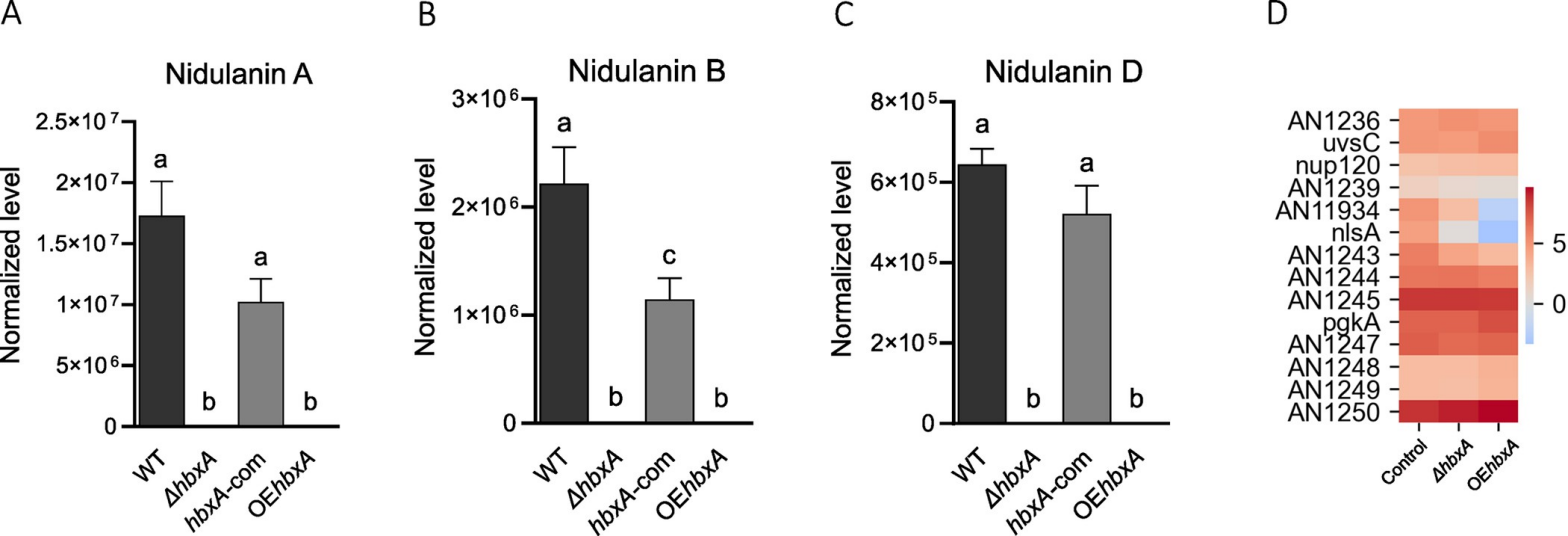
*hbxA* regulates the production of nidulanins in *A*. *nidulans*. Wild-type (WT), deletion (Δ*hbxA*), complementation (*hbxA-com*) and overexpression (OE*hbxA*) strains were top-agar inoculated on solid glucose minimum medium (GMM) at 37°C for 72 h. Samples were collected, extracted with chloroform and analyzed by LC-HRMS in positive mode **(A-C)** Quantification of nidulanin A (*m*/*z* 604.34943), B (*m*/*z* 620.34404), D (*m*/*z* 536.28659) respectively. **(D)** Heat map of TPM values of nidulanin cluster (DEGs) expression in *A*. *nidulans* Δ*hbxA* and OE*hbxA* with respect to wild type strain on a log scale found in Inglis et al. [50]. The TPM value of each gene was calculated by averaging all the TPM values of all replicates.

To gain further understanding of the effect of *hbxA* on SM in *A*. *nidulans*, as part of our transcriptome analysis, we identified DEGs in SM gene clusters and analyzed concomitant production of secondary metabolites by a metabolomics approach. Our study revealed that production of nidulaninA, nidulanin B and nidulanin D are *hbxA*- dependent (Fig 7A–7C). Both, deletion and overexpression of *hbxA*, completely inhibited the production of these compounds. In addition, the Heatmap shown in Fig 7D indicates downregulation of some of the genes in the nidulanin cluster [77], including the NRPS coding gene, *nlsA*, in both *ΔhbxA* and OE*hbxA*. This reduction in *nlsA* expression was particularly notable in the latter.

**Fig 8:**
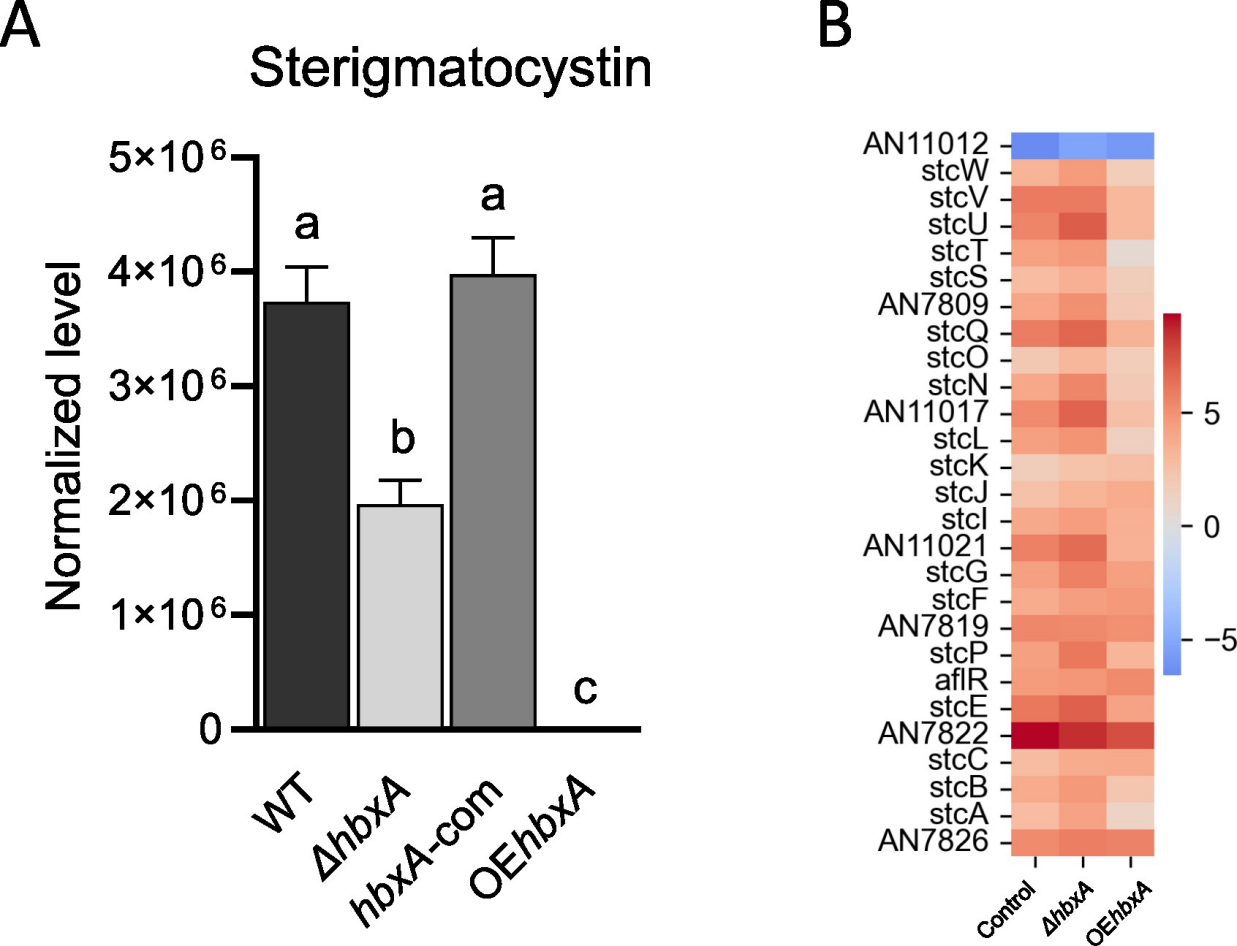
*hbxA* regulates the production of ST in *A*. *nidulans*. Wild type (WT), deletion (Δ*hbxA*), complementation (*hbxA-com*) and overexpression (OE*hbxA*) strains were top-agar inoculated on solid glucose minimum medium (GMM) at 37°C for 72 h. Samples were collected, extracted with chloroform and analyzed by LC-HRMS in positive mode. **(A)** Quantification of ST (*m*/*z* 325.07014). **(B)** Heat map of TPM values of ST cluster (DEGs) expression in *A*. *nidulans* Δ*hbxA* and OE*hbxA* with respect to wild type strain on a log scale found in Inglis et al. [50]. The TPM value of each gene was calculated by averaging all the TPM values of all replicates.

LC-MS analysis of ST confirmed the TLC results, indicating that production of this mycotoxin was reduced in *ΔhbxA* and absent in the overexpression strains (Fig 8). Unexpectedly, the Heatmap in Fig 8B shows that most of the ST genes were not downregulated in the deletion strain with respect to the wild type. However, most of the genes in this cluster were downregulated in the overexpression strain, excluding the structural genes *stcK*, *stcJ*, *stcF* and *stcC*, and the regulator, *aflR*.

**Fig 9:**
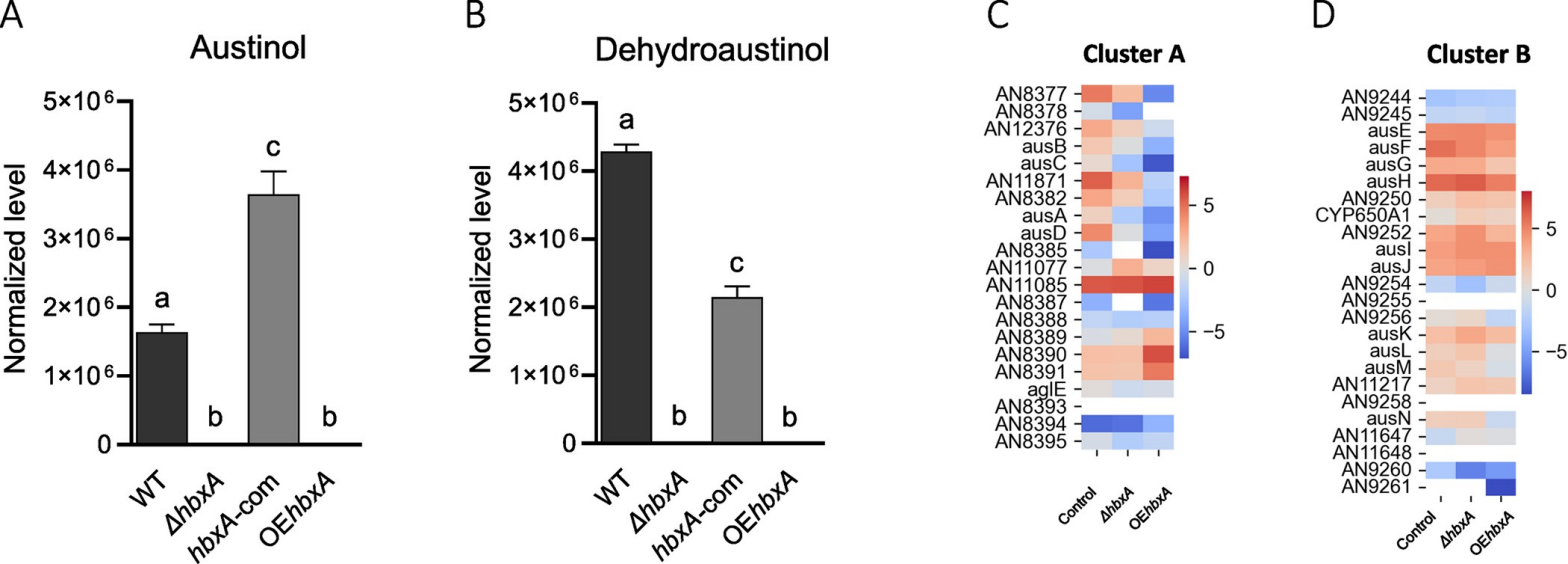
*hbxA* regulates the production of austinol and dehydroaustinol in *A*. *nidulans*. Wild type (WT), deletion (Δ*hbxA*), complementation (*hbxA-com*), and overexpression (OE*hbxA*) strains were top-agar inoculated on solid glucose minimum medium (GMM) at 37°C for 72 h. Samples were collected, extracted with chloroform and analyzed by LC-HRMS in positive mode. Quantification of **(A)** austinol (*m*/*z* 459.20059) and **(B)** dehydroaustinol (*m*/*z* 457.18524) compounds by full MS spectra resolution of 60,000 with a range of mass-to-charge ratio (*m/z*) set to 50 to 800. **(C & D)** Heatmap of TPM values of austinol cluster (DEGs) expression in *A*. *nidulans* Δ*hbxA and* OE*hbxA* with respect to wild type strain on a log scale found in Inglis et al. [50]. The TPM value of each gene was calculated by averaging all the TPM values of all replicates.

In addition, both *ΔhbxA* and OE*hbxA* strains were unable to synthesize the meroterpenoids austinol and dehydroaustinol under conditions conducive to their production in the wild type (Fig 9). The genes involved in the synthesis of these two compounds are grouped in two clusters, A and B [78]. Our transcriptome analysis revealed that most of the genes in these two clusters are downregulated in the *hbxA* deletion and also in the overexpression strains compared to the control (Fig 9C and 9D). For example, genes *ausA-D* are down regulated in both *ΔhbxA* and OE*hbxA* in gene cluster A. In cluster B, genes *ausE-G* and *ausM* are also downregulated in *ΔhbxA* and OE*hbxA*. Additionally, expression of *ausH*. *ausL* and *ausN* is reduced in OE*hbxA* with respect to the wild type.

**Fig 10:**
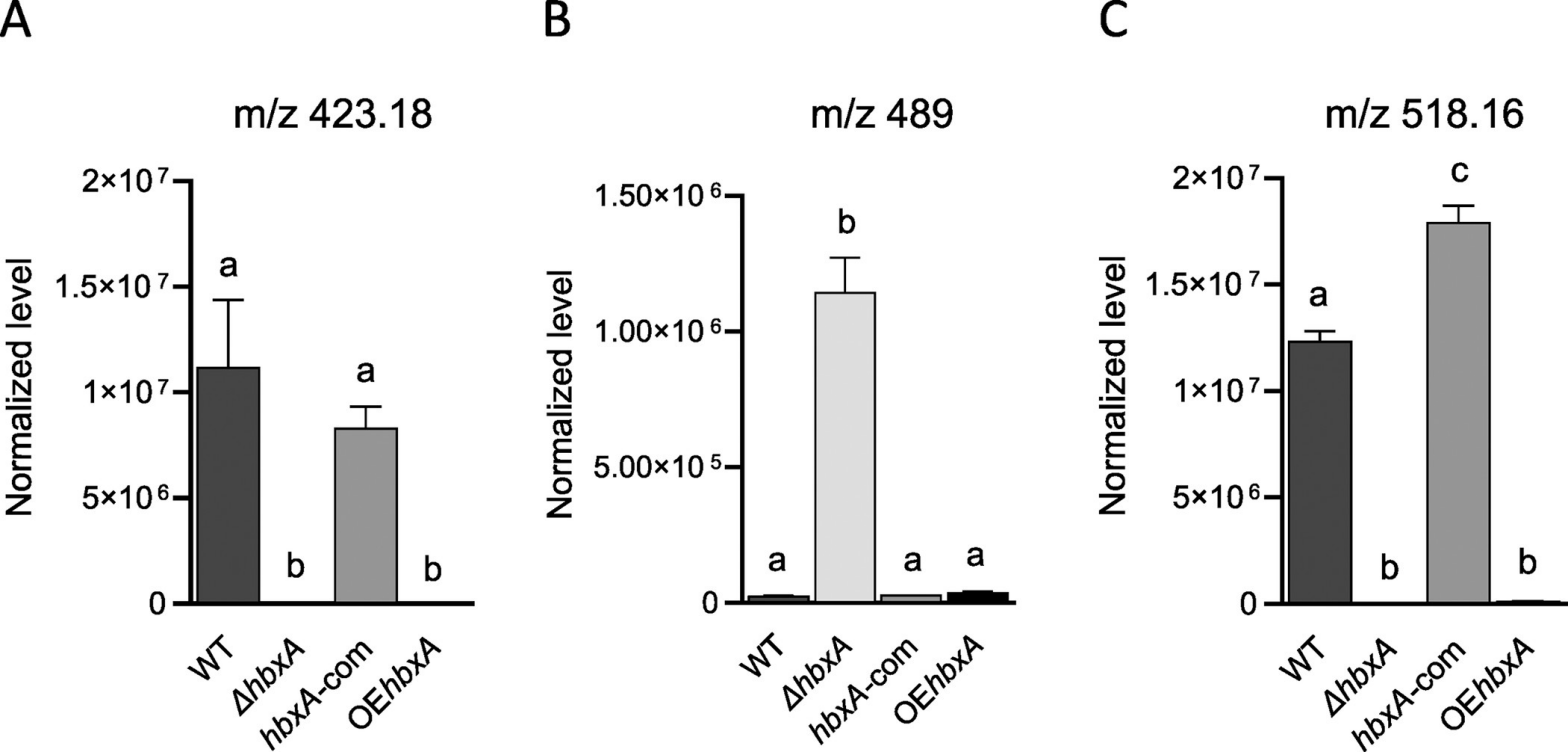
*hbxA* regulates the production of novel uncharacterized metabolites in *A*. *nidulans*. Wild type (WT), deletion (Δ*hbxA*), complementation (*hbxA-com*), and overexpression (OE*hbxA*) strains were top-agar inoculated on solid glucose minimum medium (GMM) at 37°C for 72 h, when samples were collected, extracted with chloroform and analyzed by LC-HRMS in negative mode. **(A-C)** Quantification of novel uncharacterized metabolites with *m*/*z* of 423.18012, 489.18082, and 518.16482, respectively.

Our metabolomics study also indicated that the production of three novel, unknown secondary metabolites was altered when *hbxA* was not expressed at wild-type levels. Two of these compounds (m/z 423 and m/z 518 observed in negative mode) were absent in the *hbxA* deletion strain and also in the overexpression strain (Fig 10). The third novel compound (m/z 489 in negative mode) was produced at remarkably high levels in the *hbxA* deletion strain compared to those in the wild type (Fig 10B).

## Discussion

HD-TFs have been shown to govern development in eukaryotes [13–15], including fungi [16–21]. Previous reports, together with the present study, indicate that these regulators are conserved across different fungal genera [22, 23]. In *A*. *flavus*, *hbx1*, an ortholog of *hbxA*, is also required for developmental processes, regulating genes in the conidiation central pathway, such as *brlA* and *wetA* [17, 27] as well as *flbA*, *flbC*, *flbD*, *flbE*, *fluG* and *mat1-1* [27]. In *A*. *fumigatus*, *hbxA* promotes *brlA*, *abaA* and *wetA*, as well as *flbB*, *flbD* and *fluG* expression [28]. Similarly, *hbxA* regulates conidiation in *A*. *nidulans* [33, 34]; our transcriptome study showed that *hbxA* not only regulates *brlA*, as shown in [34], but also *abaA*, *flbC* and *flbD*. These studies support that the *hbxA*-dependent regulatory mechanism of conidiation is at least in part conserved in these three Aspergillus species and possibly in other species of this genus.

Interestingly, our results revealed a broader regulatory scope for *hbxA* in *A*. *nidulans*, with more than one thousand DEGs when *hbxA* was deleted or overexpressed in this model organism, including numerous transcription factor genes. This was also the case for *A*. *flavus hbx1* [27]. However, most of the DEGs in *A*. *nidulans* are not DEGs in *A*. *flavus*; only a small percentage of homologs where DEGs in the *hbxA* and *hbx1* mutants with respect to the controls. This suggests that although the conservation of some of the regulatory mechanisms controlling conidiation appears conserved, a great part of its regulatory input is specialized in different fungal species.

Some of the TF genes involved in governing development that were found *hbxA*-dependent also control secondary metabolism in *A*. *nidulans*, for example, *mtfA* [37], *urdA* [60], *sclB* [61], *osaA* [62] and *velB* [63]. Furthermore, FunCat functional enrichment analysis showed that the category of secondary metabolism-related processes was, by far, the most enriched in *A*. *nidulans*. Our study showed that in *A*. *nidulans*, numerous genes in SM gene clusters were regulated by *hbxA*. The secondary metabolism category was also enriched in *A*. *flavus* [27]. However, the wide variation of biosynthetic gene clusters across fungal species, even in those phylogenetically close [79] could explain that although the major functional category is the same in both species, namely SM, the percentage of differentially expressed homologs is low. For example, *A*. *flavus hbx1* regulates genes in the aflatoxin, cyclopiazonic acid, aflatrem, asparasone, piperazine, and aflavarin gene clusters [27], while in *A*. *nidulans*, our study shows that *hbxA* controls genes in other gene clusters such as those responsible for the synthesis of nidulanins A, B and D, austinol and dehydroaustinol. *A*. *nidulans* HbxA also control genes in the ST gene cluster, which is partially conserved with that of aflatoxin in *A*. *flavus*. The regulatory pattern was similar; absence of both *hbxA* and *hbx1* resulted in a reduction of toxin production [17, 27]. In *A*. *flavus* deletion of *hbx1* downregulated *aflR* and other genes in the aflatoxin gene cluster. However, this was not the case in *A*. *nidulans*, suggesting that the lower levels of ST in the deletion strain, verified by both TLC and LC-MS, could be due to other factor(s). Our study showed *veA* expression is *hbxA*-dependent. VeA is a global regulator that orchestrates numerous biological processes in fungi [25, 26], such as development and SM. VeA has been shown to regulate the production of aflatoxisomes in *A*. *parasiticus* [80]. It is possible that *hbxA*, in a *veA*-dependent manner, could also influence compartmentalization of ST production in *A*. *nidulans*. This reduction in ST in the deletion strain, contrast with a previous report [34] where an increase in ST was described. It is possible that different experimental conditions in both studies could have resulted in different outcomes. Nevertheless, the most striking result is the effect of *hbxA* overexpression on ST biosynthesis as well as on the production of other metabolites. The complete elimination of ST production by *hbxA* overexpression was, in this case, accompanied by the downregulation of genes in the ST gene cluster. However, this downregulation of ST genes was, as in the case of the deletion strain, not mediated by changes in *aflR* expression.

Our study revealed that *hbxA* regulates key genes in the nidulanin gene cluster and, consequently, affects the production of the cyclic tetrapeptides nidulinins A, B and D. These compound are found in *Aspergillus* and *Penicillium* species. The function of nidulanins is not yet known. As in the case of ST, both deletion or overexpression of *hbxA* resulted in reduction or elimination of nidulinins A, B and D production, suggesting that, as in the case of VeA, certain balanced stoichiometry with respect to other regulatory factors could be needed for proper function, perhaps also interacting with other regulatory proteins. One of the genes downregulated in both deletion *hbxA* and overexpression *hbxA* strains is *nlsA*, encoding a non-ribosomal peptide synthase necessary for the synthesis of nidulanin. This enzyme has been shown to also be involved in the synthesis of fungisporin [81], which presents antibacterial activity [82], however fungisporin was not detected in our study under the conditions tested.

LC-MS indicated that *hbxA* also controls austinol and dehydroaustinol production. These are two meroterpenoids produced from polyketide and terpenoid precursors. Both austinol and dehydroaustinol have been shown to inhibit the neuraminidase enzyme, suggesting a potential for the development of new antiviral drugs [83]. Austinol also showed antibacterial activity [84]. Alteration of wild-type *hbxA* transcription by deletion or forced overexpression also resulted in a lack of production of these compounds, further supporting the possibility of a necessary stoichiometry with other regulatory partners. Two separate gene clusters, A and B [78, 84], are required for the synthesis of these compounds. Both deletion and overexpression of *hbxA* showed profound changes in the expression profile of both gene clusters, with numerous downregulated structural genes, including the polyketide synthase gene *ausA*. The prenyltransferase gene *ausN* was also downregulated in the overexpression strain.

In addition, our metabolomics analysis indicated that *A*. *nidulans hbxA* also controls the production of three unknown novel compounds. Synthesis of two of these metabolites (m/z &423 and m/z 528) did not occur in the absence of *hbxA* or when this gene was overexpressed, while the third novel compound (m/z 489) was produced at strikingly high levels in the *hbxA* deletion strain. The identity, the association with MS gene clusters, or bioactive properties of these compounds are still known and will be the subject of future studies.

Regarding additional roles of *hbxA* in *A*. *nidulans*, besides those in development and SM, our FunCat functional enrichment analysis also indicated a possible role in primary metabolism, with enrichment in the carbon-compound and carbohydrate metabolism category, particularly in the *hbxA* overexpression strain. Upregulation of the carbon catabolite repressor TF gene *creA* [66] was observed in this strain. *creA* is also under *hbx1* regulation in *A*. *flavus* [27]. Other *A*. *nidulans hbxA*-dependent regulatory genes involved in primary metabolism were, for example, *galR*, which regulates the D-galactose catabolic pathway [65], and *glcD*, which has a putative role in protein dimerization with and activation of *areB*, involved in nitrogen metabolism [64]. Other enriched categories were detoxification, virulence and disease factors and defense, suggesting its possible involvement in pathogenesis. This agrees with the fact that the *hbxA* homolog in *A*. *fumigatus* was shown to affect virulence in *A*. *fumigatus* [28].

In conclusion, we have shown that the regulatory TF gene *hbxA* governs the expression of hundreds of genes in *A*. *nidulans*, modulating not only developmental genes, but also multiple regulatory pathways. Consequently, *hbxA* governs different important aspects of this fungus’ biology, including a remarkable role in SM, regulating expression of several SM gene clusters and natural product biosynthesis, including some novel compounds. Additionally, genes associated with other cellular processes such as primary metabolisms, as well as defense and virulence, are also influenced by *hbxA*. Interestingly, a functional conservation exists between *hbxA* homologs in other *Aspergillus* species and possibly in other fungi.

## Supporting information

S1 Fig Multiple sequence alignment of *A*. *nidulans* HbxA with other fungal homologs. The HbxA deduced amino acid sequences were aligned using clustalOmega(https://www.ebi.ac.uk/Tools/msa/clustalo/). Data was visualized with boxshade using ENDscript server (https://espript.ibcp.fr/ESPript/cgi-bin/ESPript.cgi) [85] https://doi.org/10.1093/nar/gku316.
(PDF)

S2 Fig Generation of *A*. *nidulans hbxA* deletion, complementation and overexpression strains. Confirmation of the deletion (Δ*hbxA*), complementation (*hbxA*-com) and overexpression (OE*hbxA*) by diagnostic PCR. **(A)** The diagram shows replacement of *hbxA* with the marker gene *pyrG* by a double cross-over event. Primers P#2154/SD3 and P#963 were used for the diagnostic PCR, obtaining the predicted 3.01 kb product. **(B)** Schematic representation showing reintroduction of the wild-type *hbxA* allele at the *hbxA* locus in the deletion strain TSSP38.1. PCR with primers P#2154/SD3 and P#2962 confirmed the reintroduction of *hbxA* in the selected deletion strain; the expected 3.96 kb product was obtained. **(C)** Linear diagram of *hbxA* overexpression plasmid pSSP34.1. The overexpression transformant was confirmed by PCR with primers 2093 and 2218, which yielded the predicted 3.16 kb product.
(PDF)

S1 Table *hbxA*-dependent differentially expressed genes. (XLSX)

S2 Table Differentially expressed transcription factor genes. (XLSX)

## References

[1] Caesar LK, Kelleher NL, Keller NP. In the fungus where it happens: History and future propelling *Aspergillus nidulans* as the archetype of natural products research. Fungal Genet Biol. 2020 Nov 1;144:103477. 33035657 10.1016/j.fgb.2020.103477 PMC7726033

[2] Oiartzabal-Arano E, Perez-de-Nanclares-Arregi E, Espeso EA, Etxebeste O. Apical control of conidiation in *Aspergillus nidulans*. Curr Genet. 2016 May 1;62(2):371–7. 26782172 10.1007/s00294-015-0556-0

[3] Yu JH. Regulation of Development in *Aspergillus nidulans* and *Aspergillus fumigatus*. Mycobiology. 2010 Dec 31;38(4):229–37. 23956662 10.4489/MYCO.2010.38.4.229 PMC3741515

[4] Etxebeste O, Garzia A, Espeso EA, Ugalde U. *Aspergillus nidulans* asexual development: making the most of cellular modules. Trends Microbiol. 2010 Dec;18(12):569–76. 21035346 10.1016/j.tim.2010.09.007

[5] Hermann TE, Kurtz MB, Champe SP. Laccase localized in hulle cells and cleistothecial primordia of *Aspergillus nidulans*. J Bacteriol. 1983 May;154(2):955–64. 6341366 10.1128/jb.154.2.955-964.1983 PMC217550

[6] Han KH. Molecular Genetics of *Emericella nidulans* Sexual Development. Mycobiology. 2009 Sep;37(3):171–82. 23983529 10.4489/MYCO.2009.37.3.171 PMC3749384

[7] Bussink HJ, Osmani SA. A cyclin‐dependent kinase family member (PHOA) is required to link developmental fate to environmental conditions in *Aspergillus nidulans* | The EMBO Journal [Internet]. 1998 [cited 2022 Dec 12]. Available from: https://www.embopress.org/doi/full/10.1093/emboj/17.14.3990 10.1093/emboj/17.14.3990 PMC1170733 9670015

[8] Clutterbuck AJ. A mutational analysis of conidial development in *Aspergillus nidulans*. Genetics. 1969 Oct 1;63(2):317–27. 5366214 10.1093/genetics/63.2.317 PMC1212347

[9] Han KH, Han KY, Yu JH, Chae KS, Jahng KY, Han DM. The *nsdD* gene encodes a putative GATA-type transcription factor necessary for sexual development of *Aspergillus nidulans*. Mol Microbiol. 2001 Jul 1;41(2):299–309. 11489119 10.1046/j.1365-2958.2001.02472.x

[10] Kirk KE, Morris NR. The *tubB* alpha-tubulin gene is essential for sexual development in *Aspergillus nidulans*. Genes Dev. 1991 Nov 1;5(11):2014–23. 1936991 10.1101/gad.5.11.2014

[11] Lee Kim, S., Kim S. J., Han D. M., Jahng K. Y, Chae K. S. The *lsdA* gene is necessary for sexual development inhibition by a salt in *Aspergillus nidulans* | SpringerLink [Internet]. 2001 [cited 2022 Dec 12]. Available from: https://link.springer.com/article/10.1007/s002940100206 10.1007/s002940100206 11453253

[12] Miller KY, Toennis TM, Adams TH, Miller BL. Isolation and transcriptional characterization of a morphological modifier: the *Aspergillus nidulans* stunted (*stuA*) gene. Mol Gen Genet MGG. 1991 Jun 1;227(2):285–92. 2062309 10.1007/BF00259682

[13] Holland PWH. Evolution of homeobox genes. Wiley Interdiscip Rev Dev Biol. 2013 Feb;2(1):31–45. doi: 10.1002/wdev.78 23799629

[14] Mukherjee K, Brocchieri L, Bürglin TR. A comprehensive classification and evolutionary analysis of plant homeobox genes. Mol Biol Evol. 2009 Dec;26(12):2775–94. doi: 10.1093/molbev/msp201 19734295 PMC2775110

[15] Svingen T, Tonissen KF. Hox transcription factors and their elusive mammalian gene targets. Heredity. 2006 Aug;97(2):88–96. doi: 10.1038/sj.hdy.6800847 16721389

[16] Antal Z, Rascle C, Cimerman A, Viaud M, Billon-Grand G, Choquer M, et al. The Homeobox *BcHOX8* Gene in *Botrytis Cinerea* Regulates Vegetative Growth and Morphology. PLoS ONE [Internet]. 2012 Oct 25 [cited 2018 Nov 29];7(10). Available from: https://www.ncbi.nlm.nih.gov/pmc/articles/PMC3485016/10.1371/journal.pone.0048134 PMC3485016 23133556

[17] Cary, Harris-Coward P, Scharfenstein L, Mack B, Chang PK, Wei Q, et al. The *Aspergillus flavus* Homeobox Gene, *hbx1*, Is Required for Development and Aflatoxin Production. Toxins [Internet]. 2017 Oct 12 [cited 2020 Apr 17];9(10). Available from: http://www.mdpi.com/2072-6651/9/10/315 10.3390/toxins9100315 PMC5666362 29023405

[18] Arnaise S, Zickler D, Poisier C, Debuchy R. *pah1*: a homeobox gene involved in hyphal morphology and microconidiogenesis in the filamentous ascomycete *Podospora anserina*. Mol Microbiol. 2001 Jan;39(1):54–64. 11123688 10.1046/j.1365-2958.2001.02163.x

[19] Coppin E, Berteaux-Lecellier V, Bidard F, Brun S, Ruprich-Robert G, Espagne E, et al. Systematic deletion of homeobox genes in *Podospora anserina* uncovers their roles in shaping the fruiting body. PloS One. 2012;7(5):e37488. 22662159 10.1371/journal.pone.0037488 PMC3360767

[20] Kim S, Park SY, Kim KS, Rho HS, Chi MH, Choi J, et al. Homeobox Transcription Factors Are Required for Conidiation and Appressorium Development in the Rice Blast Fungus *Magnaporthe oryzae*. Copenhaver GP, editor. PLoS Genet. 2009 Dec 4;5(12):e1000757. 19997500 10.1371/journal.pgen.1000757 PMC2779367

[21] Liu W, Xie S, Zhao X, Chen X, Zheng W, Lu G, et al. A homeobox gene is essential for conidiogenesis of the rice blast fungus *Magnaporthe oryzae*. Mol Plant-Microbe Interact MPMI. 2010 Apr;23(4):366–75. 20192824 10.1094/MPMI-23-4-0366

[22] Zheng W, Zhao X, Xie Q, Huang Q, Zhang C, Zhai H, et al. A Conserved Homeobox Transcription Factor Htf1 Is Required for Phialide Development and Conidiogenesis in *Fusarium* Species. Yu JH, editor. PLoS ONE. 2012 Sep 21;7(9):e45432. 23029006 10.1371/journal.pone.0045432 PMC3448628

[23] Ghosh AK, Wangsanut T, Fonzi WA, Rolfes RJ. The *GRF10* homeobox gene regulates filamentous growth in the human fungal pathogen *Candida albicans*. FEMS Yeast Res. 2015 Dec;15(8):fov093. 26472755 10.1093/femsyr/fov093 PMC4705307

[24] Calvo AM, Wilson RA, Bok JW, Keller NP. Relationship between Secondary Metabolism and Fungal Development. Microbiol Mol Biol Rev. 2002 Sep 1;66(3):447–59. doi: 10.1128/MMBR.66.3.447-459.2002 12208999 PMC120793

[25] Calvo AM, Lohmar JM, Ibarra B, Satterlee T. 18 Velvet Regulation of Fungal Development. In: Wendland J, editor. Growth, Differentiation and Sexuality [Internet]. Cham: Springer International Publishing; 2016 [cited 2020 Apr 17]. p. 475–97. Available from: http://link.springer.com/10.1007/978-3-319-25844-7_18

[26] Calvo AM, Cary JW. Association of fungal secondary metabolism and sclerotial biology. Front Microbiol. 2015;6:62. doi: 10.3389/fmicb.2015.00062 25762985 PMC4329819

[27] Cary, Entwistle S, Satterlee T, Mack BM, Gilbert MK, Chang PK, et al. The Transcriptional Regulator Hbx1 Affects the Expression of Thousands of Genes in the Aflatoxin-Producing Fungus *Aspergillus flavus*. G3 Bethesda Md. 2019;9(1):167–78. 30425054 10.1534/g3.118.200870 PMC6325891

[28] Satterlee T, Nepal B, Lorber S, Puel O, Calvo AM. The Transcriptional Regulator HbxA Governs Development, Secondary Metabolism, and Virulence in *Aspergillus fumigatus*. Appl Environ Microbiol [Internet]. 2020 Jan 21 [cited 2020 Apr 22];86(3). Available from: https://aem.asm.org/content/86/3/e01779-19 10.1128/AEM.01779-19 PMC6974638 31757831

[29] Lim FY, Ames B, Walsh C, Keller N. Coordination between BrlA regulation and secretion of the oxidoreductase FmqD directs selective accumulation of fumiquinazoline C to conidial tissues in *Aspergillus fumigatus*. Cell Microbiol. 2014 Aug;16(8):1267–83. 24612080 10.1111/cmi.12284 PMC4114987

[30] Coyle CM, Kenaley SC, Rittenour WR, Panaccione DG. Association of ergot alkaloids with conidiation in *Aspergillus fumigatus*. Mycologia. 2007;99(6):804–11. 18333504 10.3852/mycologia.99.6.804

[31] Gauthier T, Wang X, Sifuentes Dos Santos J, Fysikopoulos A, Tadrist S, Canlet C, et al. Trypacidin, a Spore-Borne Toxin from *Aspergillus fumigatus*, Is Cytotoxic to Lung Cells. Filler SG, editor. PLoS ONE. 2012 Feb 3;7(2):e29906. 22319557 10.1371/journal.pone.0029906 PMC3272003

[32] Yao J, Jiao R, Liu C, Zhang Y, Yu W, Lu Y, et al. Assessment of the Cytotoxic and Apoptotic Effects of Chaetominine in a Human Leukemia Cell Line. Biomol Ther. 2016 Mar;24(2):147–55. 10.4062/biomolther.2015.093 PMC4774495 26902083

[33] Pandit S. S., Satterlee T., Nepal B., Lorber S., Puel O., Espeso E. A., et al. The transcriptional regulatory HbxA governs development and secondary metabolism in *Aspergillus nidulans* and *Aspergillus fumigatus*. 30th Fungal Genetics Conference, March 12–17, 2019 at Asilomar Conference Grounds in Pacific Grove, CA; 2019.

[34] Son SH, Son YE, Cho HJ, Chen W, Lee MK, Kim LH, et al. Homeobox proteins are essential for fungal differentiation and secondary metabolism in *Aspergillus nidulans*. Sci Rep. 2020 Dec;10(1):6094. 32269291 10.1038/s41598-020-63300-4 PMC7142095

[35] Käfer E. Meiotic and Mitotic Recombination in *Aspergillus* and Its Chromosomal Aberrations. In: Caspari EW, editor. Advances in Genetics [Internet]. Academic Press; 1977 [cited 2018 Oct 16]. p. 33–131. Available from: http://www.sciencedirect.com/science/article/pii/S006526600860245X 10.1016/s0065-2660(08)60245-x 327767

[36] Feng X, Ramamoorthy V, Pandit SS, Prieto A, Espeso EA, Calvo AM. *cpsA* regulates mycotoxin production, morphogenesis and cell wall biosynthesis in the fungus *Aspergillus nidulans*. Mol Microbiol. 2017 Jul 1;105(1):1–24. 28370587 10.1111/mmi.13682 PMC5506848

[37] Ramamoorthy V, Dhingra S, Kincaid A, Shantappa S, Feng X, Calvo AM. The Putative C2H2 Transcription Factor MtfA Is a Novel Regulator of Secondary Metabolism and Morphogenesis in *Aspergillus nidulans*. PLOS ONE. 2013 Sep 16;8(9):e74122. 24066102 10.1371/journal.pone.0074122 PMC3774644

[38] Szewczyk E, Nayak T, Oakley CE, Edgerton H, Xiong Y, Taheri-Talesh N, et al. Fusion PCR and gene targeting in *Aspergillus nidulans*. Nat Protoc. 2006 Dec;1(6):3111–20. 17406574 10.1038/nprot.2006.405

[39] Stinnett SM, Espeso EA, Cobeño L, Araújo-Bazán L, Calvo AM. *Aspergillus nidulans* VeA subcellular localization is dependent on the importinα carrier and on light. Mol Microbiol. 2007 Jan;63(1):242–55. 17163983 10.1111/j.1365-2958.2006.05506.x

[40] Krueger F. Trim Galore [Internet]. 2022 [cited 2022 Dec 11]. Available from: https://github.com/FelixKrueger/TrimGalore

[41] Wood DE, Lu J, Langmead B. Improved metagenomic analysis with Kraken 2. Genome Biol. 2019 Nov 28;20(1):257. doi: 10.1186/s13059-019-1891-0 31779668 PMC6883579

[42] Basenko EY, Pulman JA, Shanmugasundram A, Harb OS, Crouch K, Starns D, et al. FungiDB: An Integrated Bioinformatic Resource for Fungi and Oomycetes. J Fungi Basel Switz. 2018 Mar 20;4(1):39. doi: 10.3390/jof4010039 30152809 PMC5872342

[43] Bushnell B, Rood J, Singer E. BBMerge—Accurate paired shotgun read merging via overlap. PloS One. 2017;12(10):e0185056. doi: 10.1371/journal.pone.0185056 29073143 PMC5657622

[44] Kim D, Paggi JM, Park C, Bennett C, Salzberg SL. Graph-based genome alignment and genotyping with HISAT2 and HISAT-genotype. Nat Biotechnol. 2019 Aug;37(8):907–15. doi: 10.1038/s41587-019-0201-4 31375807 PMC7605509

[45] Li H, Handsaker B, Wysoker A, Fennell T, Ruan J, Homer N, et al. The Sequence Alignment/Map format and SAMtools. Bioinforma Oxf Engl. 2009 Aug 15;25(16):2078–9. doi: 10.1093/bioinformatics/btp352 19505943 PMC2723002

[46] Pertea M, Pertea GM, Antonescu CM, Chang TC, Mendell JT, Salzberg SL. StringTie enables improved reconstruction of a transcriptome from RNA-seq reads. Nat Biotechnol. 2015 Mar;33(3):290–5. doi: 10.1038/nbt.3122 25690850 PMC4643835

[47] Love MI, Huber W, Anders S. Moderated estimation of fold change and dispersion for RNA-seq data with DESeq2. Genome Biol. 2014;15(12):550. doi: 10.1186/s13059-014-0550-8 25516281 PMC4302049

[48] Priebe S, Kreisel C, Horn F, Guthke R, Linde J. FungiFun2: a comprehensive online resource for systematic analysis of gene lists from fungal species. Bioinforma Oxf Engl. 2015 Feb 1;31(3):445–6. doi: 10.1093/bioinformatics/btu627 25294921 PMC4308660

[49] Lind AL, Wisecaver JH, Smith TD, Feng X, Calvo AM, Rokas A. Examining the Evolution of the Regulatory Circuit Controlling Secondary Metabolism and Development in the Fungal Genus *Aspergillus*. PLOS Genet. 2015 Mar 18;11(3):e1005096. 25786130 10.1371/journal.pgen.1005096 PMC4364702

[50] Inglis DO, Binkley J, Skrzypek MS, Arnaud MB, Cerqueira GC, Shah P, et al. Comprehensive annotation of secondary metabolite biosynthetic genes and gene clusters of *Aspergillus nidulans*, *A*. *fumigatus*, *A*. *niger* and *A*. *oryzae*. BMC Microbiol. 2013 Apr 26;13(1):91. 10.1186/1471-2180-13-91 PMC3689640 23617571

[51] Etxebeste O. Transcription Factors in the Fungus *Aspergillus nidulans*: Markers of Genetic Innovation, Network Rewiring and Conflict between Genomics and Transcriptomics. J Fungi. 2021 Jul 25;7(8):600. 10.3390/jof7080600 PMC8396895 34436139

[52] Lohmar JM, Puel O, Cary JW, Calvo AM. The *Aspergillus flavus rtfA* Gene Regulates Plant and Animal Pathogenesis and Secondary Metabolism. Appl Environ Microbiol. 2019 Mar 6;85(6):e02446–18. 30635379 10.1128/AEM.02446-18 PMC6414392

[53] Arnaud MB, Cerqueira GC, Inglis DO, Skrzypek MS, Binkley J, Chibucos MC, et al. The Aspergillus Genome Database (AspGD): recent developments in comprehensive multispecies curation, comparative genomics and community resources. Nucleic Acids Res. 2012 Jan;40(Database issue):D653–9. doi: 10.1093/nar/gkr875 22080559 PMC3245136

[54] Adams TH, Boylan MT, Timberlake WE. *brlA* is necessary and sufficient to direct conidiophore development in *Aspergillus nidulans*. Cell. 1988 Jul 29;54(3):353–62. 3293800 10.1016/0092-8674(88)90198-5

[55] Andrianopoulos A, Timberlake WE. The *Aspergillus nidulans abaA* gene encodes a transcriptional activator that acts as a genetic switch to control development. Mol Cell Biol. 1994 Apr;14(4):2503–15. 8139553 10.1128/mcb.14.4.2503-2515.1994 PMC358618

[56] Chang YC, Timberlake WE. Identification of *Aspergillus brlA* response elements (BREs) by genetic selection in yeast. Genetics. 1993 Jan 1;133(1):29–38. 8417986 10.1093/genetics/133.1.29 PMC1205295

[57] Prade RA, Timberlake WE. The *Aspergillus nidulans brlA* regulatory locus consists of overlapping transcription units that are individually required for conidiophore development. EMBO J. 1993 Jun;12(6):2439–47. 8508769 10.1002/j.1460-2075.1993.tb05898.x PMC413479

[58] Etxebeste O, Ni M, Garzia A, Kwon NJ, Fischer R, Yu JH, et al. Basic-Zipper-Type Transcription Factor FlbB Controls Asexual Development in Aspergillus nidulans. Eukaryot Cell. 2008 Jan;7(1):38–48. doi: 10.1128/EC.00207-07 17993569 PMC2224158

[59] Son YE, Cho HJ, Lee MK, Park HS. Characterizing the role of Zn cluster family transcription factor ZcfA in governing development in two *Aspergillus* species. PLoS ONE. 2020 Feb 4;15(2):e0228643. 32017793 10.1371/journal.pone.0228643 PMC6999877

[60] Pandit SS, Lohmar JM, Ahmed S, Etxebeste O, Espeso EA, Calvo AM. UrdA Controls Secondary Metabolite Production and the Balance between Asexual and Sexual Development in *Aspergillus nidulans*. Genes [Internet]. 2018 Nov 23 [cited 2020 Apr 20];9(12). Available from: https://www.ncbi.nlm.nih.gov/pmc/articles/PMC6316066/10.3390/genes9120570 PMC6316066 30477161

[61] Thieme KG, Gerke J, Sasse C, Valerius O, Thieme S, Karimi R, et al. Velvet domain protein VosA represses the zinc cluster transcription factor SclB regulatory network for *Aspergillus nidulans* asexual development, oxidative stress response and secondary metabolism. PLOS Genet. 2018 Jul 25;14(7):e1007511. 30044771 10.1371/journal.pgen.1007511 PMC6078315

[62] Alkahyyat F, Ni M, Kim SC, Yu JH. The WOPR Domain Protein OsaA Orchestrates Development in *Aspergillus nidulans*. PLOS ONE. 2015 Sep 11;10(9):e0137554. 26359867 10.1371/journal.pone.0137554 PMC4567300

[63] Bayram O, Krappmann S, Ni M, Bok JW, Helmstaedt K, Valerius O, et al. VelB/VeA/LaeA complex coordinates light signal with fungal development and secondary metabolism. Science. 2008 Jun 13;320(5882):1504–6. doi: 10.1126/science.1155888 18556559

[64] Arst HN, Hondmann DHA, Visser J. A translocation activating the cryptic nitrogen regulation gene areB inactivates a previously unidentified gene involved in glycerol utilisation in *Aspergillus nidulans* | SpringerLink [Internet]. 1990 [cited 2022 Aug 23]. Available from: https://link.springer.com/article/10.1007/BF00315805 10.1007/BF00315805 2259335

[65] Kowalczyk JE, Gruben BS, Battaglia E, Wiebenga A, Majoor E, de Vries RP. Genetic Interaction of *Aspergillus nidulans galR*, *xlnR* and *araR* in Regulating D-Galactose and L-Arabinose Release and Catabolism Gene Expression. PLoS ONE. 2015 Nov 18;10(11):e0143200. 26580075 10.1371/journal.pone.0143200 PMC4651341

[66] Strauss J, Horvath HK, Abdallah BM, Kindermann J, Mach RL, Kubicek CP. The function of CreA, the carbon catabolite repressor of *Aspergillus nidulans*, is regulated at the transcriptional and post-transcriptional level. Mol Microbiol. 1999 Apr;32(1):169–78. 10216870 10.1046/j.1365-2958.1999.01341.x

[67] Askenazi M, Driggers EM, Holtzman DA, Norman TC, Iverson S, Zimmer DP, et al. Integrating transcriptional and metabolite profiles to direct the engineering of lovastatin-producing fungal strains. Nat Biotechnol. 2003 Feb;21(2):150–6. doi: 10.1038/nbt781 12536215

[68] Ukil L, Varadaraj A, Govindaraghavan M, Liu HL, Osmani SA. Copy Number Suppressors of the *Aspergillus nidulans nimA1* Mitotic Kinase Display Distinctive and Highly Dynamic Cell Cycle-Regulated Locations. Eukaryot Cell. 2008 Dec;7(12):2087–99. 18931041 10.1128/EC.00278-08 PMC2593184

[69] Dyer PS, O’Gorman CM. Sexual development and cryptic sexuality in fungi: insights from *Aspergillus* species. FEMS Microbiol Rev. 2012 Jan 1;36(1):165–92. 22091779 10.1111/j.1574-6976.2011.00308.x

[70] Pyrzak W, Miller KY, Miller BL. Mating Type Protein Mat1-2 from Asexual Aspergillus fumigatus Drives Sexual Reproduction in Fertile *Aspergillus nidulans*. Eukaryot Cell. 2008 Jun;7(6):1029–40. 18245277 10.1128/EC.00380-07 PMC2446662

[71] Piłsyk S, Natorff R, Sieńko M, Skoneczny M, Paszewski A, Brzywczy J. The *Aspergillus nidulans metZ* gene encodes a transcription factor involved in regulation of sulfur metabolism in this fungus and other Eurotiales. Curr Genet. 2015 May;61(2):115–25. 25391366 10.1007/s00294-014-0459-5

[72] Xiong Y, Wu VW, Lubbe A, Qin L, Deng S, Kennedy M, et al. A fungal transcription factor essential for starch degradation affects integration of carbon and nitrogen metabolism. PLOS Genet. 2017 May 3;13(5):e1006737. doi: 10.1371/journal.pgen.1006737 28467421 PMC5435353

[73] Ahmed YL, Gerke J, Park HS, Bayram Ö, Neumann P, Ni M, et al. The Velvet Family of Fungal Regulators Contains a DNA-Binding Domain Structurally Similar to NF-κB. Stock AM, editor. PLoS Biol. 2013 Dec 31;11(12):e1001750. 24391470 10.1371/journal.pbio.1001750 PMC3876986

[74] Röhrig J, Yu Z, Chae KS, Kim JH, Han KH, Fischer R. The *Aspergillus nidulans* Velvet-interacting protein, VipA, is involved in light-stimulated heme biosynthesis. Mol Microbiol. 2017;105(6):825–38. 28657694 10.1111/mmi.13739

[75] Alaminos Ramos. The methionine biosynthetic pathway from homoserine in *Pseudomonas putida* involves the *metW*, *metX*, *metZ*, *metH* and *metE* gene products—PubMed [Internet]. 2001 [cited 2023 Feb 16]. Available from: https://pubmed.ncbi.nlm.nih.gov/11479715/10.1007/s002030100293 11479715

[76] Chiang YM, Szewczyk E, Davidson AD, Entwistle R, Keller NP, Wang CCC, et al. Characterization of the *Aspergillus nidulans* Monodictyphenone Gene Cluster. Appl Env Microbiol. 2010 Apr 1;76(7):2067–74. 20139316 10.1128/AEM.02187-09 PMC2849234

[77] Andersen MR, Nielsen JB, Klitgaard A, Petersen LM, Zachariasen M, Hansen TJ, et al. Accurate prediction of secondary metabolite gene clusters in filamentous fungi. Proc Natl Acad Sci U S A. 2013 Jan 2;110(1):E99–107. doi: 10.1073/pnas.1205532110 23248299 PMC3538241

[78] Lo HC, Entwistle R, Guo CJ, Ahuja M, Szewczyk E, Hung JH, et al. Two separate gene clusters encode the biosynthetic pathway for the meroterpenoids austinol and dehydroaustinol in *Aspergillus nidulans*. J Am Chem Soc. 2012 Mar 14;134(10):4709–20. 22329759 10.1021/ja209809t PMC3350773

[79] Rokas A, Mead ME, Steenwyk JL, Raja HA, Oberlies NH. Biosynthetic gene clusters and the evolution of fungal chemodiversity. Nat Prod Rep. 2020 Jul 1;37(7):868–78. doi: 10.1039/c9np00045c 31898704 PMC7332410

[80] Chanda A, Roze LV, Kang S, Artymovich KA, Hicks GR, Raikhel NV, et al. A key role for vesicles in fungal secondary metabolism. Proc Natl Acad Sci. 2009 Nov 17;106(46):19533–8. doi: 10.1073/pnas.0907416106 19889978 PMC2773199

[81] Ali, Marco I Ries, Peter P Lankhorst, Rob A M van der Hoeven, Olaf L Schouten, Marek Noga, et al. A non-canonical NRPS is involved in the synthesis of fungisporin and related hydrophobic cyclic tetrapeptides in *Penicillium chrysogenum*—PubMed [Internet]. 2014 [cited 2023 Feb 16]. Available from: https://pubmed.ncbi.nlm.nih.gov/24887561/10.1371/journal.pone.0098212 PMC4041764 24887561

[82] Himaja M., Elizabeth L., Moonjit D., Asif K. Synthesis and antimicrobial evaluation of N-methylated analog of fungisporin. Univ J Pharm. 2015;4:10–4.

[83] Gozari M, Alborz M, El-Seedi HR, Jassbi AR. Chemistry, biosynthesis and biological activity of terpenoids and meroterpenoids in bacteria and fungi isolated from different marine habitats. Eur J Med Chem. 2021 Jan 15;210:112957. doi: 10.1016/j.ejmech.2020.112957 33160760

[84] Matsuda Y, Awakawa T, Mori T, Abe I. Unusual chemistries in fungal meroterpenoid biosynthesis. Curr Opin Chem Biol. 2016 Apr 1;31:1–7. doi: 10.1016/j.cbpa.2015.11.001 26610189

[85] Robert X, Gouet P. Deciphering key features in protein structures with the new ENDscript server. Nucleic Acids Res. 2014 Jul 1;42(W1):W320–4. doi: 10.1093/nar/gku316 24753421 PMC4086106

