# Supplementary material for: Homeobox transcription factor HbxA influences expression of over one thousand genes in the model fungus Aspergillus nidulans
Source: PLoS One. 2023 Jul 21;18(7):e0286271. doi: 10.1371/journal.pone.0286271 (PMC10361519; doi:10.1371/journal.pone.0286271)
Supplement: S1 Fig — The HbxA deduced amino acid sequences were aligned using clustalOmega(https://www.ebi.ac.uk/Tools/msa/clustalo/). Data was visualized with boxshade using ENDscript server (https://espript.ibcp.fr/ESPript/cgi-bin/ESPript.cgi) [85] https://doi.org/10.1093/nar/gku316. (PDF) [file pone.0286271.s001.pdf]

```
                            1        10        20         30         40
Aspergillus_nidulans        MNYIHHP.YPFAGHPSVPMEQH.LAYD........TTMVHPTMMHH........PMDGYY
Aspergillus_niger           MNYLHHP.YAFTGHAAVPMEQP.VAFD........PTMAHPSMM.H........PMDGYL
Aspergillus_flavus          MNYLHHP.YAYAGHAAVPMEQP.IAYD........PTMAHPSMM.H........PMEGYI
Aspergillus_oryzae          MNYLHHP.YAYAGHAAVPMEQP.IAYD........PTMAHPSMM.H........PMEGYI
Penicilliopsis_zonata       MSYLHHS.YPYSSHPAVPLDQP.LVFD........PAMAHSSMIPQ........PMDGYL
Penicillium_rubens          MSYIHPS.WNYQGHQGIPMDQH.MAYD........PSMVPPPMM.H........PIDGYM
Talaromyces_marneffei       MNYLHHPAYPYGVHAGIHLDQP.GLVH........P....AIA.N........NIDGYV
Blastomyces_dermatitidis    MSYLHHP.FPFGG.HAIPVDQP.VGYG........VPIRHPQLG.H........SVDTYL
Histoplasma_capsulatum      MSYLHHP.FPFSGHHAIPVDQP.FDYR........VPIRHPQLS.H........PVDTYL
Microsporum_canis           MNYLQAP.YQY....SVPMDQP.MGYG........MPISHHPYD.Q........PMDQCY
Trichophyton_tonsurans      MNYLQTP.FQY....AMPMDQP.MGYG........MPVSHHPYE.Q........PMDQCY
Ascosphaera_apis            MNYLHNP.YGYGAPTGLPIDQQQMPYG........VPIHPHPHP.HPDYHHHVPGMEPFY
Arthrobotrys_flagrans       MEV.....LSYDNKGSMPSQNPNVPVRSDVHVTQGPSPQHLNVPRHP.AANSGPHIETFI
Alternaria_alternata        ............................................................
```

```
                                    50        60        70        80        90       100
Aspergillus_nidulans        YAQPPFDMVDYYHQ..PMMDYEEYAENLSRPRLTKEQVETLEAQFQAHPKPSSNVKRQLA
Aspergillus_niger           YPHPPFDMVDFYHQ..PIMDYEEYAENLSRPRLTKEQVETLEAQFQAHPKPSSNVKRQLA
Aspergillus_flavus          YPHPPFDMIDFYHQ..PIMDYEEYAENLSRPRLTKEQVETLEAQFQAHPKPSSNVKRQLA
Aspergillus_oryzae          YPHPPFDMIDFYHQ..PIMDYEEYAENLSRPRLTKEQVETLEAQFQAHPKPSSNVKRQLA
Penicilliopsis_zonata       YPHPPFEMVDFYPP..PIMDYDEYAENLSRPRLTKEQVETLETQFQTHPKPSSNVKRQLA
Penicillium_rubens          YPHPPMEMIDYYHQ..PIMDYDEYTENLSRPRLTKEQVETLEAQFQAHPKPSSNVKRQLA
Talaromyces_marneffei       LTRPAYELADYYTHMPLMEDYEEYSENLSRPRLTKEQVDTLEAQFQAHPKPNSNKKRELA
Blastomyces_dermatitidis    LPNAPIDLADYYHQAALLEDFEQYTENLSRPRLTKQQVDTLEAQFQAHPKPNSNVKRQLA
Histoplasma_capsulatum      LPNAPIDLAEYYHQAAALEDFEEYTENLSRPRLTKDQVDTLEAQFQAHPKPNSNVKRQLA
Microsporum_canis           VPYHQNDIHGFYTT.GAIEEYEEYVENLSRPRLTKEQVEILESQFQAHPKPNSNTKRQLA
Trichophyton_tonsurans      IPYHQNDIHGFYTT.GAIEEYEEYVENLSRPRLTKEQVEILESQFQAHPKPNSNTKRQLA
Ascosphaera_apis            GHHVPVELANSFYQAVTL....EDTESLSRPRLTKEQVDTLEAQFQAHPKPNSSVKRQLA
Arthrobotrys_flagrans       GNHFPV..IPRFTP.PVSGPNEESVKEPRRFKCSREQTLALERLYQQCPKPNQATKRDLA
Alternaria_alternata        .....MDSSGGSTT...APSAPKQPGSDVKPRLTKDQHDILEQHFLAQHKPSTNVKKEFA
```

```
                                      110       120         130       140        150
Aspergillus_nidulans        QQTHLSLPRVANWFQNRRAKAKQQKRQEE..YERMQKAKAEAEE.AAKRKSESS.VPE.S
Aspergillus_niger           AQTNLSLPRVANWFQNRRAKAKQQKRQEE..FEKMQKAKAEAEE.AARGKSEST.ESS.D
Aspergillus_flavus          AQTNLSLPRVANWFQNRRAKAKQQKRQEE..FERMQKAKTEAEE.AARIKIENA.EKS.E
Aspergillus_oryzae          AQTNLSLPRVANWFQNRRAKAKQQKRQEE..FERMQKAKTEAEE.AARIKIENA.EKS.E
Penicilliopsis_zonata       AQTNLSLPRVANWFQNRRAKAKQQKRQEE..FERMQKAKAQAEE.AARGKSDTA.DQP.E
Penicillium_rubens          AQTNLSLPRVANWFQNRRAKAKQQKRQEE..FEKMTKAKAEAEE.AARRKSETL.DQL.S
Talaromyces_marneffei       VQTNLSLPRVANWFQNRRAKAKQQKRQEE..FERMQREAKEKED.QSKSIKDEEQDYGLP
Blastomyces_dermatitidis    AQTNLTLPRVANWFQNRRAKAKQQKRQEE..FERMQASEKDEQW.QNNDAPQK......E
Histoplasma_capsulatum      AQTNLTLPRVANWFQNRRAKAKQQKRQEE..FERMQASGNGEQW.KNNDTKQK......E
Microsporum_canis           LQTSLTLPRVANWFQNRRAKAKQQKRQEE..FEKMQAKEKAMAAEGSENQQQS.....E
Trichophyton_tonsurans      LQTSLTLPRVANWFQNRRAKAKQQKRQEE..FEKMQAKEKAMAAEEAESKQ.SA.....E
Ascosphaera_apis            MQTNLTLPRVANWFQNRRAKAKQQKRQAE..YEKKLAEEKAEKEQNGK..........E
Arthrobotrys_flagrans       HAINLSPTRVNIWFQNRRHRAKKHKEIQE..AKMAQILETAGRERMAKIALGTPMAL..S
Alternaria_alternata        TRLGVPLDKINNWFQNRRAKVKQDRKKLMNQYNMTMSLPFGHSHVPAM..........S
```

```
                               160       170         180                     190
Aspergillus_nidulans        SDSQRSAEAKDEKKQD..DSKAPTPKP.S......KPASDDQ.......KQSEAPAESNH
Aspergillus_niger           SK....EDAKDSKDET..DKDTPKQSV.E......NTAERTK......TPA.PSSSRPKH
Aspergillus_flavus          SN....P...DVKEET..DKETPKQSS.D......QTMSDDR......TKTPASNSRSKH
Aspergillus_oryzae          SN....P...DVKEET..DKETPKQSS.D......QTMSDDR......TKTPASNSRSKH
Penicilliopsis_zonata       SGSTVKAETPDESSNA..S.LAPQKKSAE...TTSSTASTSR.....SHVPATSAPRSRH
Penicillium_rubens          GSRKGSI...ANEESE..KSATPKQTP.T......STS.SGH......AKTDSTSSRSKH
Talaromyces_marneffei       GCDQKSPIHKDDNSHGTTKSPTPTQAS........NYTKDRP.....QTSDSSSLSRPKH
Blastomyces_dermatitidis    GES.......KQQSELPESSTTPTQRPAS...ISSC............SSPLSPAKQEEQ
Histoplasma_capsulatum      GAS.......KEQSERLESSATPTQQPDP...SSSS............LNPSE.VEKEKQ
Microsporum_canis           SSDEQQKSEQDQKNSI....LTN..TRGA....................TSSQGEHG
Trichophyton_tonsurans      SSDEQQRSEQDQKSSM....STPTDTRGA...................SSSCSEQEDHG
Ascosphaera_apis            GESKEKQESQPQESQTNE..AEATQAA..............................
Arthrobotrys_flagrans       ES...........S.M.SDLETPTSATSA...QSSLPLLTTE......FNPA....SSTS
Alternaria_alternata        NHY...A..HPQEQQHPHMLMQPDFYPNADISPASLPVQIGEGPSALDLGPQLSLQQHHH
```

```
                           200                           210        220
Aspergillus_nidulans        QQTRS................ESNRVASLAS..LQRAMDAAA..............QYQ
Aspergillus_niger           QKTRS................ESAREATFAS..LQRALNAAV..............AAR
Aspergillus_flavus          HKTKS................ESAREATFAS..LQRALNAAV..............AAR
Aspergillus_oryzae          HKTKS................ESAREATFAS..LQRALNAAV..............AAR
Penicilliopsis_zonata       QKTPS................ESAREATFAS..LQRALNAAV..............AAR
Penicillium_rubens          HKTKS................ESAREATFAS..LQRALNAAC..............AAR
Talaromyces_marneffei       QKTGS................DLAQEKTYAS..LQRAISAAV..............AAR
Blastomyces_dermatitidis    QQASD................FTTKTGCPES..PQKAMNVSM..............PSS
Histoplasma_capsulatum      QQASN................SISQPGLPEP..PQKAMKATM..............PVS
Microsporum_canis           LQTPA................EEKPEPRFDA..AGHQSKVQA.................
Trichophyton_tonsurans      LQTPA................DEEPEPKFEV..VGHTTEAQV.................
Ascosphaera_apis            .....................DKAKAAVPPQ..PQQTVNAQS..............SSE
Arthrobotrys_flagrans       TSPPY................PSSKEAAAAS..LARSITIAS..............AYA
Alternaria_alternata        QQHQHQQQQQFDMQHGLHSVPEPDRSASYRSNDLMHSIMAATNGAYMHNSGMSLNAQEP
```

```
                                         230
Aspergillus_nidulans          G.....GQGTT.................................................
Aspergillus_niger             DRYGQDGENSQ.................................................
Aspergillus_flavus            EHYSPDEQGQP.................................................
Aspergillus_oryzae            EHYSPDEQGQP.................................................
Penicilliopsis_zonata         DQFGRSGVEGEAVA.............................................P
Penicillium_rubens            DRFGRRINPRSKNE.............................................S
Talaromyces_marneffei         DQYTGPSDDHISVG.............................................P
Blastomyces_dermatitidis      AQFNQPVENNGPDR.............................................H
Histoplasma_capsulatum        AQFSQPGEDNVP.R.............................................H
Microsporum_canis             ...EVPNPEPIKLI.............................................P
Trichophyton_tonsurans        ...EIPSSEPAKMI.............................................P
Ascosphaera_apis              TRSEQPVLPRPSNNAQNG........................................QR
Arthrobotrys_flagrans         SSYEGEGVSQGYHP.............................................G
Alternaria_alternata          EFYDTTGLSNAYSSDLSAFSVPAPMPNDLAPSHPEFDNFADFQLDYSALAATNTSNSASA

                                                     240       250
Aspergillus_nidulans          ............SMGGSG..SVSPTTSLPNDA...........................D
Aspergillus_niger             ............SPSMDG..SVSPTTTFSNNRCGSHDSSRHGQGDLSSSF......S.QN
Aspergillus_flavus            ............ATIHEG..SVSPTTTYSGMNN..HGDSRAAQSSSTTPFSEWENAK.ET
Aspergillus_oryzae            ............ATIHEG..SVSPTTTYSGMNN..HGDSRAAQSSSTTPFSEWENAK.ET
Penicilliopsis_zonata         PPP.........ADALEG..SMPSSKMYSL........DERSQTALNPTFPEWETPAEST
Penicillium_rubens            VPEED....EEEEAVSPG..SMPPPKAVT......PANDHGNLANINSSFSGWSGIK.EE
Talaromyces_marneffei         TDR.....................AFDVRQTNIPVPSANNTPQTSANSTLSEWGSSR.DS
Blastomyces_dermatitidis      .................E..SSAITYHLSQ.....SPSGKNGGSTFTSSLSGWNGME.DN
Histoplasma_capsulatum        .................G..PSDIKYHFPQ.....SSLGNDDCGTFPSNLCGWNGIE.DN
Microsporum_canis             SPPMK......EDQEMSG..NVVD..MHN......TAQPPFSQPGVDSHFQQWG......
Trichophyton_tonsurans        SSPIK......DSQEMSG..TMTT..MHNN.....TAQPSFSRPETNGHFQQWA......
Ascosphaera_apis              SQPQSTGNVNQQNFEHHGATSVSPAISV...........NVVPDQIHVQYPEWGNAGYHQ
Arthrobotrys_flagrans         ............AAPYDG..LPPPNYSLD.........PYDPNFYPVSNFSDWGSSY.NS
Alternaria_alternata          EAQQSTGSISSDASPYNGTQSSGTTQSP.......NGPTPPSIASLNSVYTGWTEDP...

                                         260
Aspergillus_nidulans          SAVWSSVNST..NGE.........LSVPG...............................
Aspergillus_niger             AISWTSQ.SS..QGALGYVTSGESLTIPGMDGTQ.....HDAGHD..SMQ..NV......
Aspergillus_flavus            AMSWSASQSP..QEHLGYS.AAESLTVPELDGSH.....QNVQHS....D..TL......
Aspergillus_oryzae            AMSWSASQSP..QEHLGYS.AAESLTVPELDGSH.....QNVQHS....D..TL......
Penicilliopsis_zonata         SLTWTPSQSP..EDTFSYGNLNAATSFTSMVESLSVAEMETPQQS..FRR..........
Penicillium_rubens            PASWGSAE.H..NDNVGYSTTEQTSYSTSCHQAM..ADISNPSHS..MQQ..NLDVYS..
Talaromyces_marneffei         SIAWTPSQSP..EEGYEFGSLNNVPFASEVA......QMDNSPNP..DVS..TSHGFG..
Blastomyces_dermatitidis      HAIWPSPQGA..EDSFDFGHLNRHHDNPLEIRV................Q..GSPHFP..
Histoplasma_capsulatum        HAIWSSSQDI..EDHIVFPHLNKPHGNPLEICV................S..ESQQFP..
Microsporum_canis             .....PTPVP..QIEVRVGSADAHPVATSAP.IM...ELDSNGYWSPDSSGIVNQPFT..
Trichophyton_tonsurans        .....STGVP..QIGVNVGSADAHTVSTAAP.VM...EIDSNGYWSPDSSGLVSQPYT..
Ascosphaera_apis              DAGQ........PT..............................................
Arthrobotrys_flagrans         SVSYTPTPAGQREDPFEFDNMSALNS...............PYHNAPL.SG.VPPVFQDD
Alternaria_alternata          SQMAHPKQVEEPEDQFAPYSLAQASA...........SEQTLPFW............P..

                                                   270                280              290
Aspergillus_nidulans          ....................LENSQSFSDY........RSASDA...........GASYN
Aspergillus_niger             ......QF.HPSQNEDWSHPLQTSKSLSGY........RSASDA...........EVSYN
Aspergillus_flavus            ......QF.HSSQNEEWSGQVQGTKSFPGY........HSSNDA...........EASYS
Aspergillus_oryzae            ......QF.HSSQNEEWSGQVQGTKSFPGY........HSSNDA...........EASYS
Penicilliopsis_zonata         ............PSDGWSHHHHQHQHHNHHRHHHHMQEHVSKHHHP.......SASAMEV
Penicillium_rubens            ......NH.LPPHTEEWSED..................RESRHD...........NLVYS
Talaromyces_marneffei         ......SI.TSRSQQMWNPQFQGRAEIHA.........................SDPMYG
Blastomyces_dermatitidis      ......SIQFSHEPDGWGYHSMP..HLVG......EQRHNSQDS...........GDGFY
Histoplasma_capsulatum        ......CIQFNHEPGEWECHSMP..HLMG......KQRNSSQDL...........GEGFH
Microsporum_canis             .....DLLCNGSSPEELPCQVQPDTTPFS...........................QSFM
Trichophyton_tonsurans        .....ELLCNNSSPEDLSCQVQPTTTAFT...........................QSFM
Ascosphaera_apis              .........ELPFRDTWGTSFAGNGSTE............................MVSG
Arthrobotrys_flagrans         YSAFAAAVQMNSMPKAMNMNLKSRPVPS...........................GLVLK
Alternaria_alternata          ..............QDGSSQMYPQSNFY.......QQSNTSAHAILSTPEQARKLSAAPS

                                            300                 310
Aspergillus_nidulans          SMQFALQ......ADAANARRASR..........SLETGQTARPG...............
Aspergillus_niger             GVQYPLQ......QDLSLPRRGSS..........DELADTLEGIGIN......THPSS..
Aspergillus_flavus            AAQYTLH......PESSLSRRGSS..........DDLADSLEGIGIH......AA.....
Aspergillus_oryzae            AAQYTLH......PESSLSRRGSS..........DDLADSLEGIGIH......AA.....
Penicilliopsis_zonata         SFTYPS......AASMEFTRRGSS..........DDLADSLEHIGID......ATDSV..
Penicillium_rubens            NMQYPMPM...QAPDISVTRRESS..........SALTSSLEGIGIC......TTGQS..
Talaromyces_marneffei         SLSYSSLQ...PPSATSSRRPSAS..........EELADSISGIGIN......TAALA..
Blastomyces_dermatitidis      QIPFHALQ...SPLYPEEPRRDSY.....SSE.QSELADTLFHTEIN......NVIELSP
Histoplasma_capsulatum        PIPFHALQ...SPLYPDEHRRGSS.....SSE.QSELADTLFHTEIN......SMIETSP
Microsporum_canis             QSDMPLYS....SQNLVNQRHVSL.....PME.QGDI...SPRSGPN......SDSELTH
Trichophyton_tonsurans        PSELPLYS....SQSIVNQRHVSL.....PME.QGDI...SPSSGPN......SDPESTH
Ascosphaera_apis              VIPMPCT......PLMEEHEH.............GDMVSPMVNTPVSTMETPATSVQISP
Arthrobotrys_flagrans         NSDRPAFSRVATCPDLSNVESISMQQTHRPMHTSSETSSPVLENGPKQL...........
Alternaria_alternata          DLDIPLHF.....REDAFARRNSS.........SSNLANNMDAIH...............
```

```
                                         320       330       340       350
Aspergillus_nidulans          ....................KKPARSLISLPAETDR.GLPRVGTSRSTSMLS.TS..IMS
Aspergillus_niger             ....QLVN....EGD..RSSWKEPSKELDLAARRKRPRPAAIGTSRSSSMLT.GSS.TMS
Aspergillus_flavus            ....GLPI....RTD..RSSWKEAGKELDLAARRKRPRPAAIGTSRSSSMLA.GSAASMS
Aspergillus_oryzae            ....GLPI....RTD..RSSWKEAGKELDLAARRKRPRPAAIGTSRSSSMLA.GSAASMS
Penicilliopsis_zonata         ....HLNP.....QRVDPAAWKEPGKELDLAARRKRPRPAAIGTSASGRSSLAAGTVSMS
Penicillium_rubens            ....GLSQ....PVDRVEATWKEPGKELDLAARRKRPRPAAIGTSGTRPLANSTSMSSLS
Talaromyces_marneffei         ....G.........SMDSSMWRRPEKELDIAARRKRPRPAAIGTAHHRLS...TNPSMVS
Blastomyces_dermatitidis      QNTPHLNLAQLHHQAEPSTNWRYPEKEVDIAGRRKRPRPAAIGTSSMSRS...YGPSSMS
Histoplasma_capsulatum        RNTPHLNMAQLRHQVDPTTNWRYPEKEVDIAARRKRPRPAAIGTPAMSRS...YGPSSVS
Microsporum_canis             SE..G........RQLPRLHISTSDNAIGLAARRKRPRPAAIGTSGLSRAL..GGPPSMS
Trichophyton_tonsurans        PE..G........RQPPRLHISTSDNAIGLAARRKRPRPAAIGTSGFGRTV..GGPVSGS
Ascosphaera_apis              MAP.........PTVDNMDTWRQFKKEVDIAARRKKPRPAAIGTATLGRSF..TGPSSVS
Arthrobotrys_flagrans         .AAPGM........ERSQSYTERPSLQQEIALRRLRPMPPSLGPNARTQRIFHG.KINGS
Alternaria_alternata          ............IRNSTPDGFQPPDQQSSIAARRQK.RPVNLSSSAMRSASYSA..PMSS

                               360       370       380       390       400
Aspergillus_nidulans          PTTRG..QNY..GT...VKQSKSAQNLG....SRYAGVRKPS.AQRSPLNLSTFAEAGVL
Aspergillus_niger             PSTRL..PSY..GNGHAVRQSKSAQGLN....SRYAGVRKASAAQRSPLNLSTFAEAGAL
Aspergillus_flavus            PTTRL..PSY..GSAPGVRQSKSAQCLN....SRYAGVRKASAAQRSPLNLSSFAEAGAL
Aspergillus_oryzae            PTTRL..PSY..GSAPGVRQSKSAQCLN....SRYAGVRKASAAQRSPLNLSSFAEAGAL
Penicilliopsis_zonata         PTTRL..PSSLGATGHSVRQTKSAQSLN....SRYAGVRKVSVAQRSPLNFSTFAEAGAL
Penicillium_rubens            PTARM..PSS..GAGNSMRQSKSTQSLN....SRYAGVRKASAAQRSPLNFT.FADSGSI
Talaromyces_marneffei         PNARM..ATF..GAPHTIRHAKSSHTLG....SRYAGVRKLSATQRSPLGYSSFAEAATA
Blastomyces_dermatitidis      PTTRI..HGM..GAGHVLRHAKSTQNLSPSHTSRYPGIRKASAPQRSPLGITSFAEASLF
Histoplasma_capsulatum        PTTRI..QGM..GAGHILRHAKSTQNLSP...NRYPGIRKASVAQRSPLGITSFGEASRF
Microsporum_canis             PTRRV..SSA...AWGGVRKSSQLAELS....PRYASVRKLSGSPRSPFPYSLEGRQHAL
Trichophyton_tonsurans        PTRRV..SSA...AWSGVRKSSQLAELS....PRFGGMRKISGSPRSPFPYSLEGRAHAL
Ascosphaera_apis              PTLGVTRPGYGPGHCHTLRQTKSTQSLGHSARSRLSGIRKTSYNSRSPLNLSSFTENSLL
Arthrobotrys_flagrans         HS..........VHGTPLPTPPSDADFG........NATVQHKLKRKPSDLSKEHS....
Alternaria_alternata          PG.........GNGDKVIRRIRSSGIPNA..GGRV.QKSQPGSAQRSPMVSS.FSDAAAS

                                        410       420       430       440       450
Aspergillus_nidulans          S.SAKT....ELSTMLQPV.TTNSLAPPTPLTPEDLHHLLPTTPSTDGYCLSAQPTAHLF
Aspergillus_niger             G.S.KA....DMSSMLQPAVTTGGLAPPTPLTPEDLHHLLPTTPSDGGYCLSAQPTSQLF
Aspergillus_flavus            G.TSKP....EMSSMLSPAVTTGGLAPPTPLTPDDLHHFIPNTPSDGGYCLSAQPTSQLF
Aspergillus_oryzae            G.TSKP....EMSSMLSPAVTTGGLAPPTPLTPDDLHHFIPNTPSDGGYCLSAQPTSQLF
Penicilliopsis_zonata         S.AAKA.......EMLQPSVSANTLAPPTPLTPEDFQHLLPPASPEGGYCLSAHPASQQL
Penicillium_rubens            K.........KAEKMLRPSISTTSLAPPTPLTPQDLQHFMPASPTDSNYCLSAHSTAHFF
Talaromyces_marneffei         A.AANASSESRQKHRLHTSASVGNLAPPTPLTPEDFQHMLLTPTTSDTQMN..FSTPHLT
Blastomyces_dermatitidis      N.CANAT....DMMSTVPGLVTTTLAPPTPLTPEDLRTLLPPTPNDSQYCVSPTDDM...
Histoplasma_capsulatum        N.CANTA....DLMSTLPGLVTTSLAPPTPLTPEDLQTLLPPTPNDSQYCVSPTDDM...
Microsporum_canis             S.NT.........DLAVPSSTTSSIPPATPLTPDEMQYLLPPTPIDNQYCLSPQEEM...
Trichophyton_tonsurans        S.NA.........DLAVPPSTTSSIPPATPMTPDEMQYLLPPTPIDNQYCLSPQEEM...
Ascosphaera_apis              G.SALP................TISPLATPMTPDGARSLMPPTPNDAYA.YSMPECP...
Arthrobotrys_flagrans         .....................................PDVPDLESDGGF............
Alternaria_alternata          PKFART....FSTSSATTIGHGGSLAPPTPLTPQDFGNYWGGAA.....VIRPHSAMPDH

                                    460                   470       480          490
Aspergillus_nidulans          ......PTTQPMQINIAS............PPATPLGMDIMS...SYPYHSVAPPMSAPA
Aspergillus_niger             ......PTTQPMQINIAS............PPATPLAVDVLS...SYPYQGVAPPMSAPA
Aspergillus_flavus            ......PTTQPMQINIAS............PPATPMAMDMLS...TYQYHSVAPPMSAPA
Aspergillus_oryzae            ......PTTQPMQINIAS............PPATPMAMDMLS...TYQYHSVAPPMSAPA
Penicilliopsis_zonata         FQTSTTATTQPMQIHIAS............PPSTPLTMEVLS...PFAYTTLAPPLSAPA
Penicillium_rubens            ......PTSQPMQVNMAS............PPATPL..DIYS...PFPYQNVAPPMSAPA
Talaromyces_marneffei         DTQGNFPVTQSMQINVAS............PPETPLTLDVFS...AMQYQNMAPPLSATP
Blastomyces_dermatitidis      GCARFFPISQPMQVHIES............PPTTPLHLGVPS...HLQYQSMGVPMSASS
Histoplasma_capsulatum        GCARLFPMSQPVQVHIES............PPTTPLHLGVQS...HLQFQSMGVPMSTPS
Microsporum_canis             GYAHSFPTSQSMNFDENQ........ESKRQPPPFVMVGMPH...AQSYQSFTEPMSAPP
Trichophyton_tonsurans        GYSHSFPTSQSMTFDECQ........ESKRQPPPFVMVGMPH...PQSYQQFTEPMSAPP
Ascosphaera_apis              SYA..APPPTGMSLEPES............PPGTPFNFEYGMPRPSLHHTSFVPPRSAPP
Arthrobotrys_flagrans         .....TNEDAPIKQNLAS............PPPTPETAGLAAVNGSFKE......ESSDQ
Alternaria_alternata          ......NSPESMHTNWSSDQAGNVIAKTTSPPSSSLDLQSRFVND..ALYRDTPPQSAPA

                                500                  510        520       530
Aspergillus_nidulans          NFTSFP.DYS.............CD.GSFQGRNWE.ATSMPSPEVPFQSQCH....Q..M
Aspergillus_niger             HYTSFA.DYAS............CE.APLTGRSWTDATSMPSPEASFQSPCQ....LPQA
Aspergillus_flavus            HYTSFP.DYVT............CEGAPLTGRSWTGANSMPSPEAAFQNRVP....ITQA
Aspergillus_oryzae            HYTSFP.DYVT............CEGAPLTGRSWTGANSMPSPEAAFQNRVP....ITQA
Penicilliopsis_zonata         QYTTFPPEYAG............SCEVPLTARSWAEAVPMPSPEMNFAVGMP....PSIA
Penicillium_rubens            QVSSFP.EYLT............CDSVPIPARSWADTGSISSPEYPAGLQVP....HS.S
Talaromyces_marneffei         QYASFT.DYSP............ITSEPLTGVSWAVSTPDASL...FPSSLQ.....SQR
Blastomyces_dermatitidis      QRTTFQEYALS............VPTNPMNGGLWSDVSSMPSPETSHLQQPT..IHMPQP
Histoplasma_capsulatum        QHTPFQEYQLS............IPTNPMSGGHWPDASSMSSPETSHLQQPT..IHMPQP
Microsporum_canis             NFTTFNELIPD............CGQQQQQQALTSSEADPS.......LNV..IHMPRP
Trichophyton_tonsurans        NFTTFNELIPG............CGQQQQ.HHPLSSSEAETS.......LNV..IHMPRP
Ascosphaera_apis              NQHGFPDSNNF.MPIPGSAPGTAAGPHAMSDYAWSTGSS.LSPDTPHLQNSH..FSHPHT
Arthrobotrys_flagrans         QYTNFTKMLEQSSPIVSTAAFDGHSDFQPNVEHWISGHPQPYWTPHGQSDNHVNYDISQQ
Alternaria_alternata          TQQHFPRTSYMQ..............QPQMRAA.............FHSTTDLTIQQPKP
```

```
                                       540       550
                                        .         .
Aspergillus_nidulans       N.............F..SSIPYDHALDQ...................SQSENG......P
Aspergillus_niger          D.............L..TTMSYEQAMEQ...................GA...DHVPVTGS
Aspergillus_flavus         D.............V..SSLSYGQALEQ...................GRQPADSLSAAGS
Aspergillus_oryzae         D.............V..SSLSYGQALEQ...................GRQPADSLSAAGS
Penicilliopsis_zonata      Q...........HSI..SPIP.....DQ...................GL...SMD.....
Penicillium_rubens         T.............V..SPMGYNATTDH...................TGQAFGMESVSGS
Talaromyces_marneffei      Q............QP..PIYIYEQDDDEHQ.......DPKWTLSGDDGSSLYGSTKASAT
Blastomyces_dermatitidis   ............THI..SPITYEESFDSGN.......PA.........LVEDTVTSQSES
Histoplasma_capsulatum     ............THI..SPITYGESLDSGN.......PA.........LVEDMMTGQRES
Microsporum_canis          ............THI..SPVAYDDQMQGEQQVEDSAATEDWQGQGQQS.....T..QSTS
Trichophyton_tonsurans     ............THI..SPIAYDDQVQGEH.VEESAPAEEWQGQGQPS.....STHSTTG
Ascosphaera_apis           HQMLPQQHNGTAQHISPPSMTYESPFEFENNHP........MMEGMNPLSPQGTIRAASP
Arthrobotrys_flagrans      SQQAYGIHDGRQRH..............................................
Alternaria_alternata       ............EHFRRPSLPDTAQAQG.N.....DSSSQYMQAGNMHYDEFKDVSLSGI

                            560       570       580       590
                              .         .         .         .
Aspergillus_nidulans       SQSPFGD...ADI.....QAPGDASKATEFHLYEFPDQEEAHR..............FVA
Aspergillus_niger          PSLVYHT...SDVDMPTSAAFCGDSKQTEFHIYEFPEQQEAHR..............FVA
Aspergillus_flavus         PPLMYTT...DADMHTSSGSFHGDAKPTEFYIHEFPEQQEAHR..............FVA
Aspergillus_oryzae         PPLMYTT...DADMHTSSGSFHGDAKPTEFYIREFPEQQEAHR..............FVA
Penicilliopsis_zonata      ..........HETGSYSGSPPGSHAKETEFRIQEFPEQQEAHR..............FVA
Penicillium_rubens         PSLIYSI...EDTDIPGSAELA.ERKRPEFMMHEFPEHDTHFG...............GH
Talaromyces_marneffei      PPA..........NMMTVSEEHDPNGMTQFHIHEFPKQQEAHR..............NVA
Blastomyces_dermatitidis   PQCTVKSCGTPTTSSP.QGSTPGSQKVTEFLIQEFPEQQEAHR..............RAA
Histoplasma_capsulatum     PHCAVKNCNTPTASSP.QGSSPGSRRVTEFLIQEFPEQQEAHR..............RAA
Microsporum_canis          PNSPISE.......SGQGYNSTGKGASTEFYIQEFPQQDEALK..............MAA
Trichophyton_tonsurans     PNSPVSE.......SSQAYNSAGKS.NTEFYIQEFPQQDEAMK..............VAA
Ascosphaera_apis           PQMD...........QSGYPVQG...QQNFAQQSMKAQTPEFSFMQQLTGADERDDGTSD
Arthrobotrys_flagrans      ............................................................
Alternaria_alternata       H.............HN.VPFAPQVSAMPDFLVHQYTPPQGTDS............HGNLL

                              600       610
                                .         .
Aspergillus_nidulans       QQLPNQKPKAYTFADNRTPTNFG........
Aspergillus_niger          QQLPSQKPKAYTFNNQ.TPNDF.........
Aspergillus_flavus         QQLP.QKPKAYTFNNQ.TPSDWRGN......
Aspergillus_oryzae         QQLP.QKPKAYTFNNQ.TPSDWRGN......
Penicilliopsis_zonata      AQLSVQPPKKYTFINNATGHVEA........
Penicillium_rubens         HLSSMQKPKAYTFANNTTPSNYPS.......
Talaromyces_marneffei      QQLAPQIPKNYTFSNQ.TPSDF.........
Blastomyces_dermatitidis   EQLPPQKPMNYTFSNH.TPNDF.........
Histoplasma_capsulatum     EQLPPQKPMNYTFSNH.TPNDF.........
Microsporum_canis          QQLPPQRARTYTFTNQ.TPNDFYRTAIFPPI
Trichophyton_tonsurans     QQLPPQRARTYTFTNQ.TPNDFYRTAIFPPV
Ascosphaera_apis           AMLTPYDQGTYMFSTP.TTHLLA........
Arthrobotrys_flagrans      ...............................
Alternaria_alternata       RRTTEPQPKSYIFANQ.GPGDFRGQ......
```
