# Supplementary figures and images for: Homeobox transcription factor HbxA influences expression of over one thousand genes in the model fungus Aspergillus nidulans
Source: PLoS One. 2023 Jul 21;18(7):e0286271. doi: 10.1371/journal.pone.0286271 (PMC10361519; doi:10.1371/journal.pone.0286271)

A

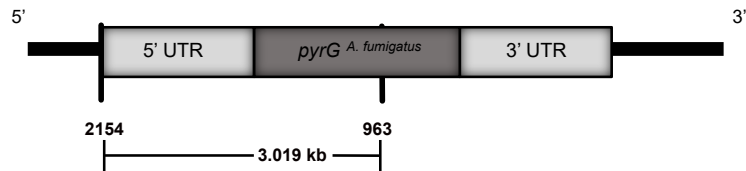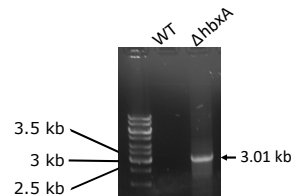

B

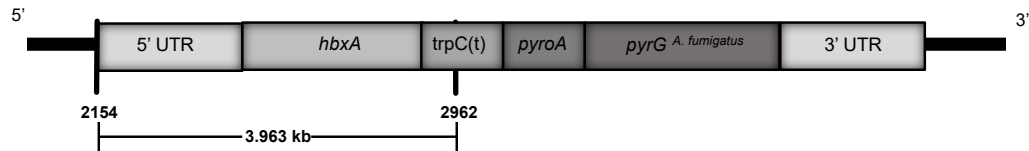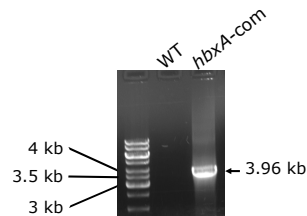

C

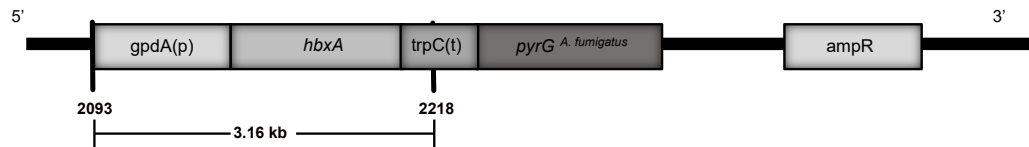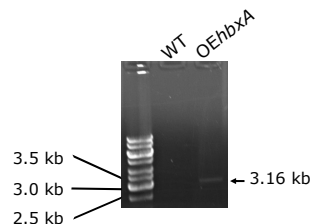

Supplement: S2 Fig — Confirmation of the deletion (ΔhbxA), complementation (hbxA-com) and overexpression (OEhbxA) by diagnostic PCR. (A) The diagram shows replacement of hbxA with the marker gene pyrG by a double cross-over event. Primers P#2154/SD3 and P#963 were used for the diagnostic PCR, obtaining the predicted 3.01 kb product. (B) Schematic representation showing reintroduction of the wild-type hbxA allele at the hbxA locus in the deletion strain TSSP38.1. PCR with primers P#2154/SD3 and P#2962 confirmed the reintroduction of hbxA in the selected deletion strain; the expected 3.96 kb product was obtained. (C) Linear diagram of hbxA overexpression plasmid pSSP34.1. The overexpression transformant was confirmed by PCR with primers 2093 and 2218, which yielded the predicted 3.16 kb product. (PDF) [file pone.0286271.s002.pdf]
